# Characterising commensal and pathogenic staphylococcal interactions with neonatal and adult blood

**DOI:** 10.1038/s41598-025-30393-8

**Published:** 2025-12-09

**Authors:** Isabella Anna Joubert, Christopher Mullally, Penghao Wang, Abha Chopra, Tobias Strunk, Andrew Currie

**Affiliations:** 1 School of Medical, Molecular & Forensic Sciences, Murdoch University, Perth, Australia; 2 Personalised Medicine Centre, Murdoch University, Perth, Australia; 3 Wesfarmers Centre of Vaccines and Infectious Diseases, The Kids Research Institute Australia, Perth, Australia; 4 Clinical Perinatal Research Laboratories, King Edward Memorial Hospital, Perth, Australia; 5 Centre for Crop and Food Innovation, Food Futures Institute, Murdoch University, Perth, Australia; 6 Murdoch Medical Genomics Core Laboratory, Precision Medicine Center, IIID, HFI, Murdoch University, Perth, Australia; 7 Neonatology, Child and Adolescent Health Service, King Edward Memorial Hospital, Perth, Australia; 8 Cooper Medical School of Rowan University, Camden, New Jersey 08103 United States

**Keywords:** (6): Neonatal sepsis, Host-pathogen interaction, Dual RNA-sequencing, Staphylococcus epidermidis, Late-onset sepsis, Preterm infant, Computational biology and bioinformatics, Immunology, Microbiology, Systems biology

## Abstract

The abundant skin commensal, *Staphylococcus epidermidis*, is the leading cause of late-onset sepsis (LOS) in preterm infants but rarely causes infections in term infants and adults. Staphylococcal virulence mechanisms and the role of the preterm immune responses in driving these life-threatening infections remain poorly understood. Using an ex vivo sepsis model, we challenged whole blood from very preterm infants (30–32 weeks gestational age, GA; *n* = 8), term infants (> 37 weeks GA; *n* = 8), and young adults (18–25 years; *n* = 8) with either live *S. epidermidis* or *S. aureus* (~ 10^7^ colony-forming units, CFU/ml) for 90 min. Dual RNA-sequencing (RNA-seq) was performed to simultaneously assess host and pathogen gene expression profiles, identifying common and pathogen-specific responses across cohorts. We found shared immune processes induced in all age groups upon bacterial challenge, including cytokine (*IL1A*,* IL1B*,* IL6*,* IFNB1*) and chemokine (*CCL20*,* CCL3*,* CCL7*,* CXCL2*) signalling. Preterm infants also exhibited unique responses, such as increased platelet activation and fibrin clot formation, Wnt signalling, and hypoxia pathways in response to *S. epidermidis* challenge. Our findings suggest that bacterial gene co-expression, including iron acquisition and heme biosynthesis genes, are also influenced by the hosts developmental age, highlighting the complexity of host-bacterial interactions in the early stages of neonatal sepsis.

**Supplementary Information:**

The online version contains supplementary material available at 10.1038/s41598-025-30393-8.

## Introduction

Very premature infants (< 32 weeks gestational age, GA) are at significantly increased risk of developing late-onset sepsis (LOS; onset > 72 h of life)^1^ due to several factors, including exposure to nosocomial pathogens, medical interventions (i.e., vascular catheters, parenteral nutrition, antibiotics), and distinct immune defences^2–4^. In high-income countries, the majority of LOS episodes are caused by Gram-positive pathogens, with coagulase-negative *Staphylococcus* spp. (CONS), particularly *Staphylococcus epidermidis*, and *S. aureus* being the most commonly isolated species from blood cultures^2,5–7^.

*S. epidermidis* and *S. aureus* colonise skin and mucosal surfaces within a few days after birth^8,9^ and can adhere to host and medical devices while evading the immune system^10,11^. These mechanisms likely facilitate bacterial invasion when host barriers are breached and/or immune functions are compromised^12,13^. However, the mechanisms underlying the occasional transition of these species from commensal/colonising behaviour to invasive pathogenesis remain incompletely understood^14,15^. Clinical outcomes in LOS depend, among other things, on the causative pathogen, with Gram-negative bacteria linked to higher morbidity and mortality^16^. LOS mortality rates due to CONS in preterm infants range from 1.9 to 9.4%, with higher rates reported for *S. aureus* (10.8–13.1%)^17,18^. Pathogen-specific host immune responses, particularly variations in pro-inflammatory mediator production, likely contribute to these differences^19–25^.

Although all preterm infants have shared risk factors for infection, the vast majority does not acquire LOS, despite nearly ubiquitous exposure to CONS and *S. aureus*^26^. This suggests that additional influences, such as environmental exposures, host-specific immune functions, and strain-specific virulence play a role in shaping host-pathogen interactions in this population. Both in vitro human and in vivo murine studies have indicated that *S. epidermidis*, despite being a common commensal species, provokes a potent and specific immune response in preterm infants^14,27,28^. However, direct comparative studies of neonatal host immune interactions with *S. epidermidis*, and more virulent staphylococcal species like *S. aureus*, remain limited^29^.

Several studies using adult whole-blood challenge models have highlighted mechanistic differences in how *S. aureus* and *S. epidermidis* interact with the host. While *S. aureus* is typically a more aggressive bloodstream pathogen, *S. epidermidis* fits the description of an “accidental” pathogen^30^, relying on stealth and metabolic adaption rather than active manipulation of host immunity. For instance, live (but not heat-killed) *S. aureus* strains actively suppressed host immunity in an ex vivo bacteraemia model, reducing chemokine levels and impairing neutrophil recruitment^31^, an effect not observed with live *S. epidermidis* in this study. *S. aureus* also induces high levels of complement and coagulation activation products in human whole blood, which may be used by the invading pathogen to favour survival^32^. By contrast, *S. epidermidis* responses in whole blood appear to focus on metabolic adaptation, such as biosynthesis, metabolism of amino acids, and iron acquisition, with less direct immune evasion^33^.

In this study, we sought to identify the distinct virulence strategies employed by *S. epidermidis* and *S. aureus* in whole blood of adults, and neonates with varying sepsis susceptibility, using an optimised ex vivo sepsis model coupled with dual RNA-sequencing transcriptomic analysis.

## Results

### Staphylococcal blood challenge model

Whole blood samples (1 ml) were collected from 8 very preterm infants, 8 term infants, and 8 young adults (Table 1). Blood samples (0.45 ml each) were challenged for 90 min with either live *S. epidermidis* or *S. aureus* or remained unchallenged (0.1 ml) to serve as a baseline control. Bacterial challenge doses were comparable for *S. epidermidis* across all host groups (Table 1). Challenge doses for *S. aureus* were similar in preterm and term infant blood samples but ~ 30–40% lower in adult blood (*p* = 0.005, compared to term samples).

**Table 1:** Demographic data of study participants and experimental bacterial challenge doses. 8 preterm infants, term infants, and adults were recruited for this study. Participant ages, sex, infant birth weights, and bacterial doses median (range) of live *S. epidermidis* and *S. aureus* used for blood challenge are listed. *, *p* < 0.005; comparing adult to term challenge doses using dunn’s test.

Variable	Preterm infants	Term infants	Adults
**n**	8	8	8
**Age (years)**	-	-	22.1 (18–25)
**Gestational age (weeks)**	31.0 (30.1–31.9)	39.1 (37.9–41.6)	-
**Birth weight (g)**	1,620 (1,120-1,998)	3,380 (2,840-4,660)	-
**Males (%)**	37.5	50	37.5
***S. epidermidis*** ** dose (CFU/ml)**	1.1 × 10^7^ (6.5 × 10^6^−2.2 × 10^7^)	1.3 × 10^7^ (8.4 × 10^6^−1.7 × 10^7^)	1.1 × 10^7^ (8.5 × 10^6^−1.3 × 10^7^)
***S. aureus*** ** dose (CFU/ml)**	1.3 × 10^7^ (8.9 × 10^6^−2.4 × 10^7^)	1.5 × 10^7^ (8.9 × 10^6^−2.4 × 10^7^)	*9.3 × 10^6^ (9.0 × 10^6^−9.7 × 10^6^)

**Fig. 1:**
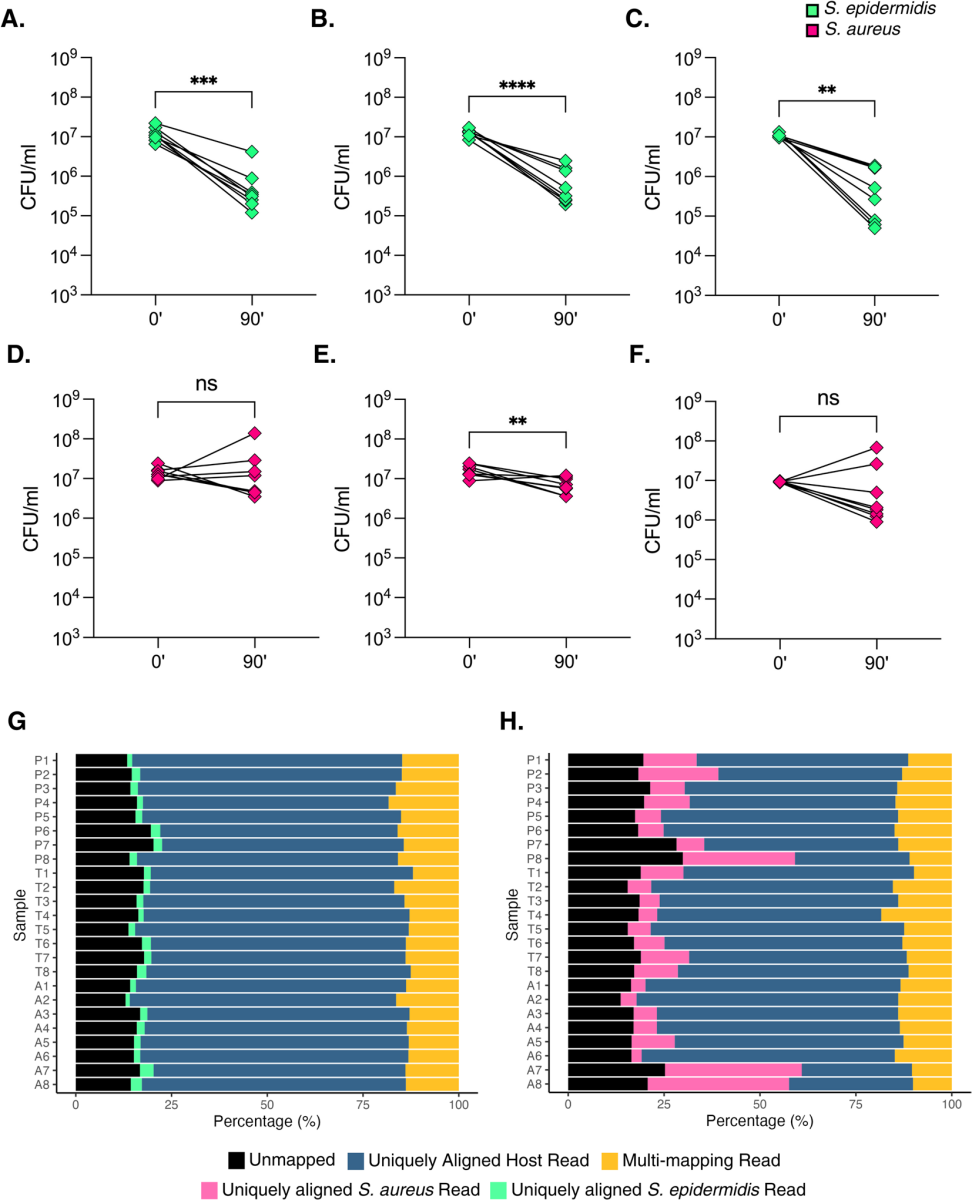
Recovery of *S. epidermidis* and *S. aureus*, total RNA, and dual host/pathogen reads after 90 min of blood challenge. **A-C**, *S. epidermidis* recovery from preterm infant, term infant, and adult blood samples. **D-E**, *S. aureus* recovery from preterm infant, term infant, and adult blood samples. Data show colony counts for each individual blood sample, comparing initial challenge inoculum to the bacterial count recovered post challenge (90 min) using paired t-test (preterm and term data) and Wilcoxon matched-pairs signed rank test (adult data). **G-H**, Distribution (%) of host, pathogen, multi-mapping, and unmapped reads across individual preterm infant (P), term infant (T), and adult (A) blood samples stimulated with *S. epidermidis* or *S. aureus*, respectively. Statistical significance shown as: *, *p* ≤ 0.05; **, *p* ≤ 0.01; ***, *p* ≤ 0.001; ****, *p* ≤ 0.0001.

Post challenge sample plating showed that *S. aureus* survived and grew in blood samples after 90 minutes, with a median recovery of 139% (IQR: 18% to 157%) above the starting inoculum in preterm infant and adult blood samples (Fig. 1D-F). However, *S. aureus* numbers significantly decreased in the term infant cohort (*p* < 0.01; Fig. 1E). This contrasted with *S. epidermidis*, where a median of only 9% (IQR: 2% to 11%) of the starting inoculum were recovered from each sample, a significant decrease in each cohort (*p* < 0.01) (Fig. 1A-C).

Post-challenge, total host and bacterial RNA was isolated and quantified. Purified RNA yields varied significantly across cohorts: term infants presented the highest RNA yields from stimulated samples, averaging 11.8 µg (range: 6.6–17.0 µg), followed by preterm infants at 6.0 µg (range: 2.8–9.5 µg), and adults at 1.5 µg (range: 0.6–3.5 µg) (Supplementary Fig. 1).

All individual RNA samples underwent dual RNA-sequencing, followed by RNA-seq read filtering and trimming. In silico read alignment showed that approximately 70% (57.8–73.2%) of reads could be uniquely mapped to either host or bacterial genomes across all samples. The remaining 30% of reads comprised primarily multi-mapping reads or reads that were too short or otherwise unsuitable for accurate mapping. Unique read counts for *S. epidermidis* ranged from 274,553 to 730,547 (2.8–9.5% of total unique reads; Fig. 1G, **Supplementary Table 1**), while *S. aureus* reads showed greater variation with 598,996 − 10,200,000 reads (7.3–74.8%) across samples (Fig. 1H, **Supplementary Table 1**). Host and bacterial read count data was filtered, normalised, and analysed separately.

### Developmental age-specific host transcriptional responses

**Fig. 2:**
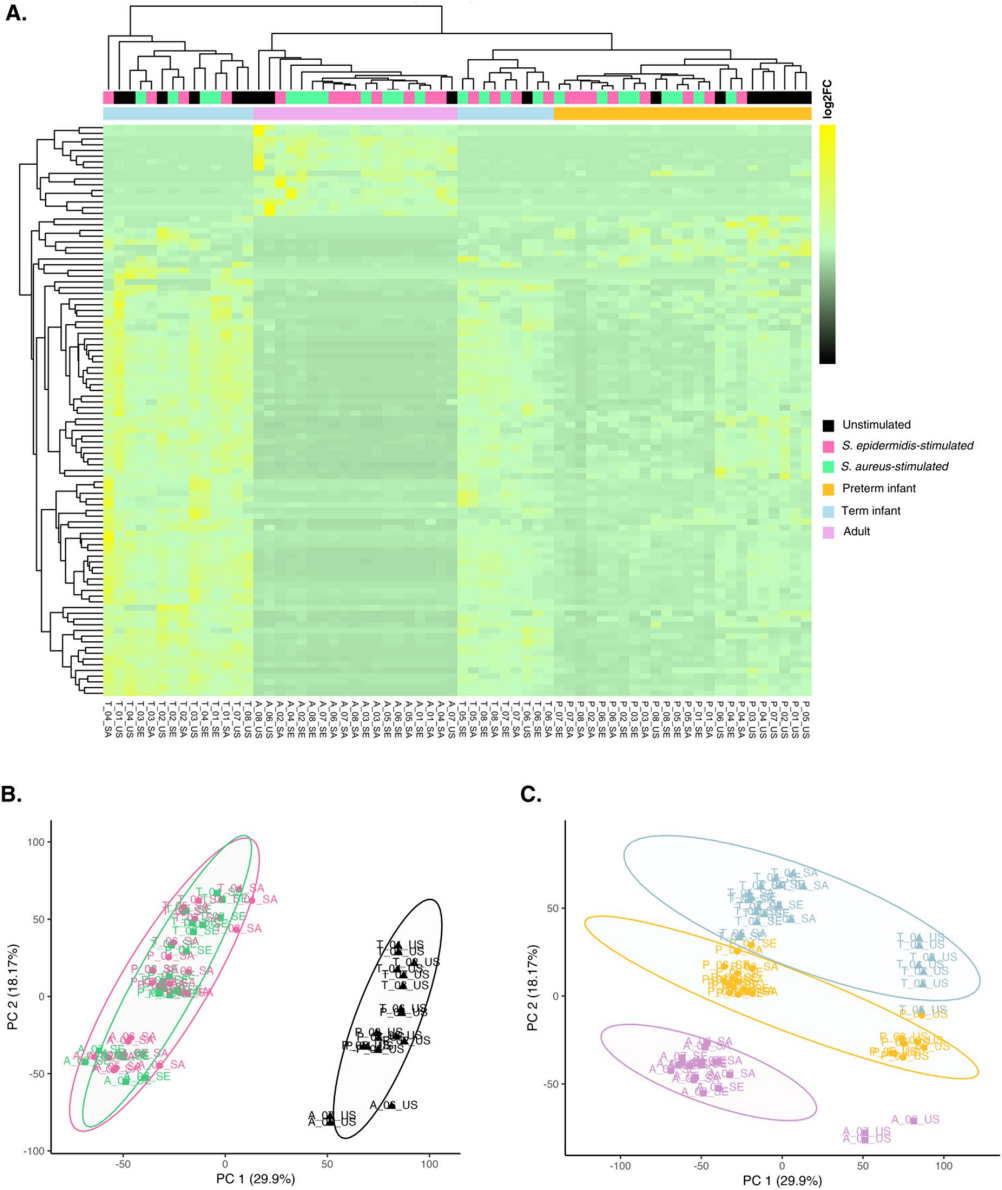
Age- and pathogen-specific host gene expression in whole blood samples. **A**. Heatmap showing the top 100 host DEGs based on absolute log2 fold change (log2FC) and adjusted p-values < 0.05 across all samples. Hierarchical clustering was performed on rows and columns using Euclidean distance and complete linkage. Colour blocks represent cohort (age) and pathogen (challenge) information for each sample. **B-C**, Principal Component Analysis (PCA) was performed on variance-stabilised host read count data. Colour ellipses represent 95% confidence regions for each “Cohort” (B) or “Pathogen” (C) group, respectively.

We conducted a DESeq2 analysis on the filtered host count matrix, identifying the top 100 differentially expressed host genes (DEGs) based on the largest absolute log2 fold change (FC) and adjusted p-values (padj < 0.05). These DEGs were visualized in a heatmap, which showed distinct clustering of samples by cohort but not by pathogen challenge (Fig. 2A). Principal Component Analysis (PCA) confirmed that preterm infant, term infant, and adult samples formed distinct clusters along PC2, which accounted for 18.17% of the data variance (Fig. 2B). Unstimulated control samples clustered separately along PC1 (29.9% variance), while samples stimulated with *S. epidermidis* and *S. aureus* exhibited significant overlap across all cohorts (Fig. 2C).

**Fig. 3:**
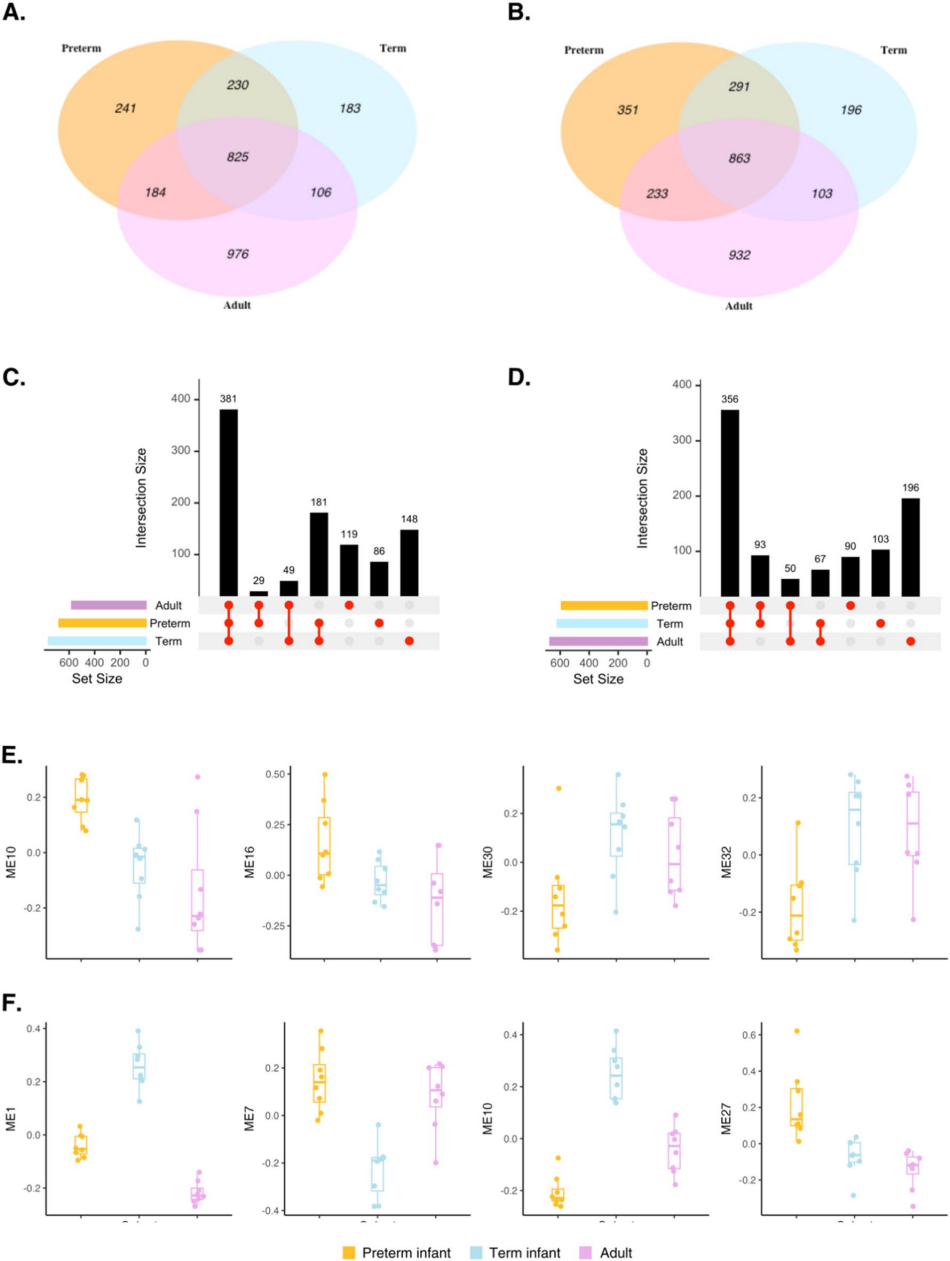
Age-specific transcriptional host responses to bacterial challenge. Venn diagrams showing the number of differentially expressed host genes (DEGs) in preterm infants, term infants, and adults in **A**. *S. epidermidis*- and **B**. *S. aureus*-challenged samples compared to unchallenged baseline control samples. UpSet plots show the interactions sizes of significantly enriched GO terms between the 3 host cohorts in **C**. *S. epidermidis*- and **D**. *S. aureus*-challenged samples. Weighted Gene Correlation Network Analysis (WGCNA) was performed to identify the 4 top-ranked co-expressed Module Eigengenes (ME) in response to **E**. *S. epidermidis*, and **F**. *S. aureus* in preterm infants, term infants, and adults, based on linear modelling, empirical Bayes smoothing and multiple testing correction. Top-ranked MEs are defined as showing the most significant differences in expression patterns between groups defined by the experimental variable (Cohort age).

We then identified all significant host DEGs in response to *S. epidermidis* and *S. aureus* when compared to unstimulated baseline control samples across each host cohort (preterm infant, term infant, and adults). 825 and 863 DEGs were shared across the three cohorts in response to both *S. epidermidis* and *S. aureus* challenge, respectively (Fig. 3A-B). Preterm infants also expressed 241 unique DEGs only in response to *S. epidermidis* and 351 unique DEGs in response to *S. aureus*. To identify the biological processes which are induced in host blood upon bacterial challenge, we performed Gene set overrepresentation analysis on the DEG lists, both shared and unique to each cohort. Many signifcantly enriched Gene Ontology (GO) terms (*p* < 0.05, q < 0.10) were induced across all cohorts in response to bacterial challenge. These included cytokine-mediated signalling, cellular responses to lipopolysaccharides and bacterial molecules, cellular responses to IL-1, T cell and leukocyte activation, and responses to chemokines and TNF (Fig. 3C-D, **Supplementary Fig. 2)**. We also identified 86 and 90 enriched GO terms which were unique to preterm infants in response to *S. epidermidis* and *S. aureus*, respectively (Fig. 3C-D). Upon *S. epidermidis* challenge, biological processes related to Wnt signalling and responses to oxygen levels and hypoxia were enriched in preterm infant samples specifically (**Supplementary Table 2**), while several processes related to neuron projection development and nervous system development were uniquely upregulated in response to *S. aureus* (**Supplementary Table 3**).

Weighted Gene Co-expression Network Analysis (WGCNA) was used to construct networks of genes connected by similarities in expression and grouped into co-expressed gene modules (Module Eigengenes, MEs). Reactome pathway and GO term analysis was then performed to identify any enriched biological processes, pathways, and molecular functions in the identified co-expressed gene modules which we found differentially expressed between neonatal and adult host cohorts.

Figure 3 depicts the top 4 MEs which differed most significantly in their co-expression between the three host cohorts across *S. epidermidis*- and *S. aureus*-challenged samples. For instance, gene modules 10 and 16 were induced at higher levels in preterm infant blood compared to term infants and adults in response to *S. epidermidis* (Fig. 3E). We found that the genes included in ME10 were enriched in processes related IL-12 family signalling, while ME 16 included genes involved in platelet activation, blood coagulation, and negative regulation of wound healing. MEs 30 and 32, on the other hand, were downregulated in preterm infants compared to the other cohorts, and we found genes with functional enrichments in IL-27-mediated signalling, interferon responses (ME 30), neutrophil degranulation, and other innate immune pathways enriched in these modules.

In response to *S. aureus*, we found MEs 7 and 27 upregulated in preterm compared to term infants (Fig. 3F). Functionally, ME 27 showed overlap with *S. epidermidis*-stimulated ME16, and was found enriched in genes involved in processes such platelet activation, signalling and aggregation, while ME 7 was enriched for TNF-mediated signalling, as well as various apoptotic processes and cellular stress responses. ME 1 was most highly induced in term infant blood upon *S. aureus* stimulation and comprises genes encoding for proteins involved in heme biosynthesis and metabolic processes, (erythrocyte) hemopoiesis, and iron ion homeostasis. A full heatmap of all identified MEs and their relative expression in each cohort can be found in **Supplementary Fig. 3**.

### Pathogen-specific host transcriptional responses

**Fig. 4:**
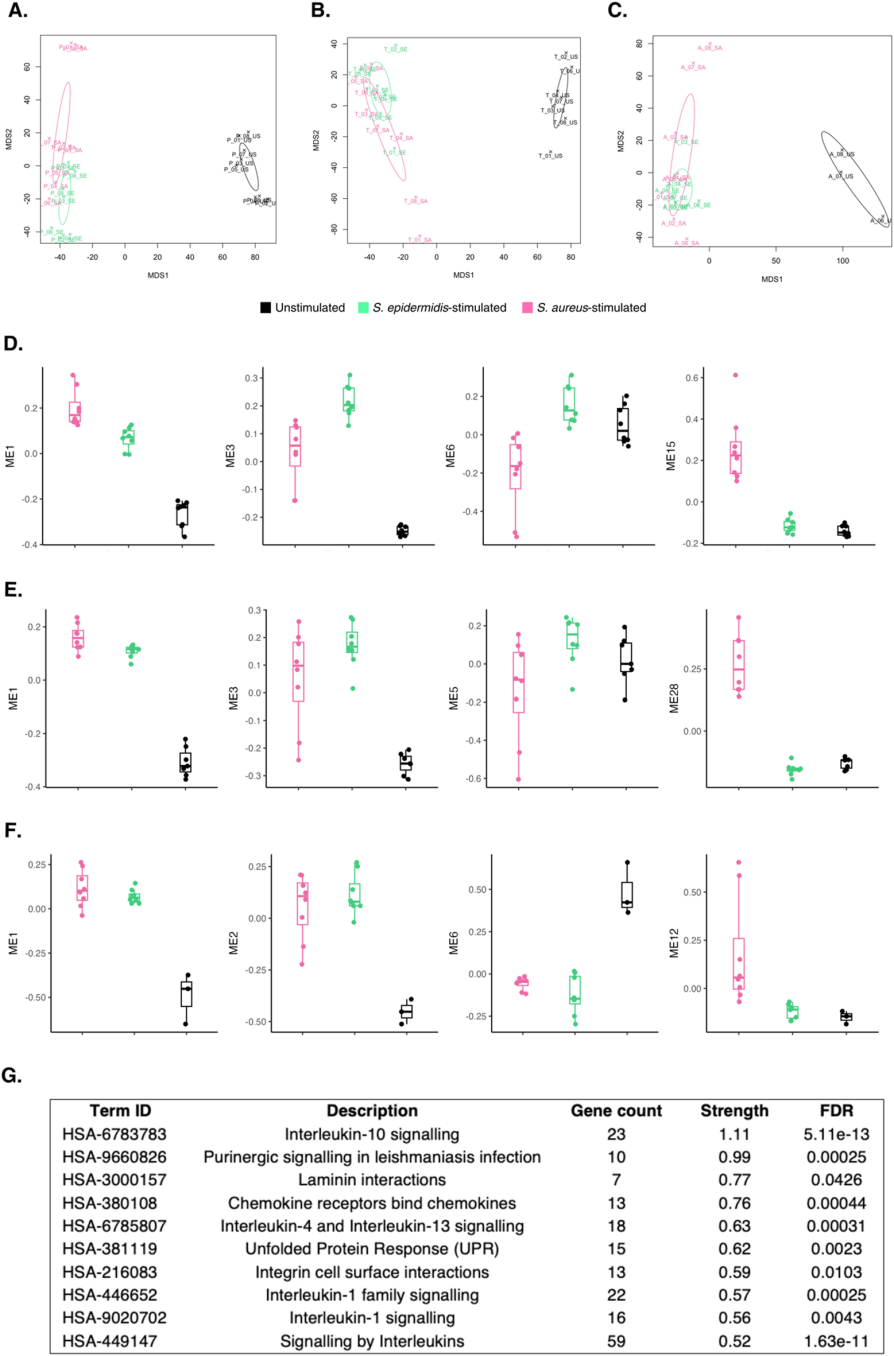
Pathogen-specific transcriptional responses in preterm infants, term infants, and adult blood. Multidimensional Scaling (MDS) plots of **A**. preterm infant, **B**. term infant, and **C**. adult samples challenged with *S. epidermidis*, *S. aureus*, or unstimulated control samples, with ellipses showing the 95% confidence interval. WGCNA analysis was used to identify the 4 top-ranked co-expressed Module Eigengenes (ME) in **D**. preterm infants, **E**. term infants, and **F**. adults, based on linear modelling, empirical Bayes smoothing and multiple testing correction. Top-ranked MEs are defined as showing the most significant differences in expression patterns between groups defined by experimental variable (Pathogen challenge). **G**. Reactome pathway enrichment analysis of 1002-gene cluster (ME3), which was identified by WGCNA as differentially expressed in response to *S. epidermidis* and *S. aureus* in preterm infants. Enrichment analysis was performed using STRING and top 10 enriched pathways are shown sorted by strength of enrichment. FDR: False Discovery Rate.

Multidimensional scaling (MDS) analysis revealed a clear separation between unstimulated and pathogen-exposed samples in all host cohorts. We observed that the transcriptional profiles between *S. epidermidis*- and *S. aureus*-challenged host blood samples showed substantial overlap (Fig. 4A-C). To identify any pathogen-specific host transcriptional responses, we compared the DEGs in response to *S. epidermidis* and *S. aureus* in each host population separately. We found the majority of DEGs (1,167 in preterm infants, 1,068 in term infants, and 1,725 in adults) were shared in response to both bacterial pathogens, however, we also identified a number of pathogen-specific DEGs in each cohort (**Supplementary Fig. 4**). GO terms uniquely enriched in preterm infants in response to *S. epidermidis* included lipid localisation and transport, response to oxygen levels and hypoxia, and multiple protein serine/threonine kinase signalling and coagulation processes. Upon *S. aureus*-challenge, regulation of neuron projection development, peptidase activity, and IL-1/IL-2 signalling processes were found significantly enriched in preterm infants (data not shown).

WGCNA was then used to identify all co-expressed gene modules in each host cohort and their expression was compared between unstimulated and pathogen-stimulated samples. The largest gene module (i.e., ME 1) in each cohort was induced in both the *S. epidermidis* and *S. aureus*-stimulated samples compared to unstimulated samples in all cohorts. We also observed MEs which showed differences in their co-expression in *S. epidermidis* and *S. aureus*-challenged samples (Fig. 4D-F). For instance, ME 3 comprises a large network of > 1,000 genes, which was induced in preterm infant blood samples in response to both bacterial pathogens, but exhibited higher levels of expression in *S. epidermidis*-challenged samples (Fig. 4D). Reactome analysis identified several enriched pathways in this ME, including IL-1, IL-3, IL-4, and IL-10-signaling (Fig. 4G). Similarly, ME 6 was expressed at higher levels in preterm infants in response to *S. epidermidis* and comprised 449 genes, which were enriched in regulation of matrix metallopeptidase secretion, TLR 2 and 4 signalling pathways, canonical glycolysis, and the positive regulation of myeloid leukocyte cytokine production involved in immune response. A full heatmap of all identified preterm and term infant MEs and their relative expression can be found in **Supplementary Fig. 5.**

### Bacterial transcriptional responses to blood challenge

**Fig. 5:**
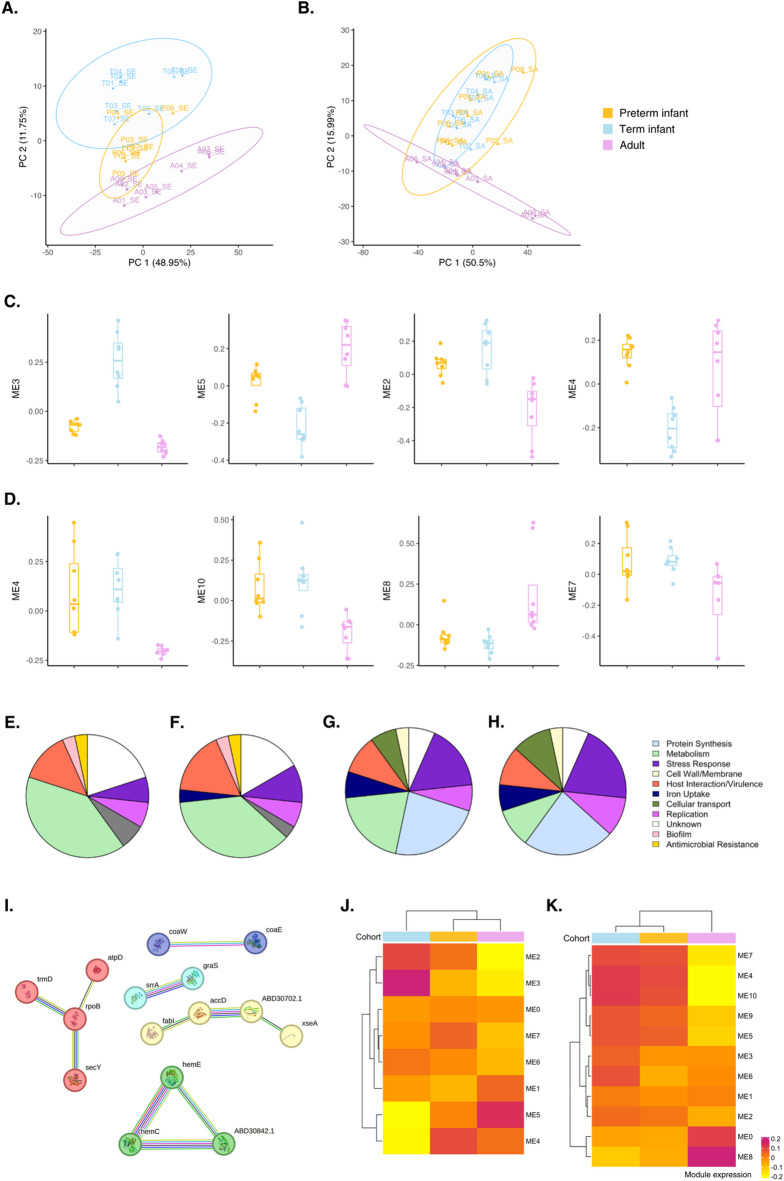
*S. epidermidis* and *S. aureus* gene expression profiles in response to whole blood. PCA analysis was performed using **A**. *S. epidermidis*, and **B**. *S. aureus* gene expression counts after variance-stabilising transformation was performed using the DESeq2 package in R. Ellipses show the 95% confidence interval. Co-expressed gene modules (Module Eigengenes, ME) in **C**. *S. epidermidis* and **D**. *S. aureus* and their relative expression in preterm infant, term infants, and adult blood samples. **E-H**. Pie charts show the distribution of functional categories across the top 30 most highly expressed **E**. *S. epidermidis* genes in preterm, **F**. *S. epidermidis* genes in term, **G**. *S. aureus* genes in preterm, and **H**. *S. aureus* genes in term blood samples. Genes were assigned to categories by manual gene annotation. **I**. STRING protein association network in *S. epidermidis* ME 4, showing connected proteins with high confidence (> 0.7). MCL clustering was performed with an inflation parameter of 3. Line colours of protein interactions (edges) indicate the type of interaction evidence. Edges between individual MCL clusters are depicted as dotted lines. Unconnected nodes were removed from the networks. STRING analysis was based on *S. epidermidis* strain RP62A. Heatmaps showing all MEs of **J**. *S. epidermidis* and **K**. *S. aureus* across host cohorts.

To investigate whether *S. epidermidis* and *S. aureus* also exhibit host-specific bacterial gene expression patterns, we normalized bacterial read counts using variance-stabilizing transformation and performed PCA (Fig. 5A-B). PCA revealed that the first two components (PC1 and PC2) explained approximately 60% and 66% of the variance in the *S. epidermidis* and *S. aureus* datasets, respectively. In *S. epidermidis*, gene expression patterns distinctly separated adult and term infant blood samples, while preterm infant responses overlapped with both cohorts (Fig. 5A). In contrast, *S. aureus* responses to preterm and term infant blood were largely similar, with less cohort-specific separation (Fig. 5B).

WGCNA of bacterial read counts identified eight and eleven co-expressed MEs in *S. epidermidis* and *S.* aureus, respectively (Fig. 5J-K). Modules were ranked by their differential expression across the 3 host cohorts, highlighting the top four modules for each pathogen (Fig. 5C-D). In *S. aureus*, most MEs were differentially expressed between adult and infant blood samples. For example, ME 8 was significantly upregulated in adult samples and included genes involved in bacterial metabolic processes such as *de novo* inosine monophosphate (IMP) biosynthesis, purine and biotin biosynthesis, and glutamine metabolism. In *S. epidermidis*, gene module co-expression varied more broadly across the three host cohorts. ME 2 and 3 were highly expressed in term infant blood, while ME 4 and 5 were downregulated in term infant blood compared to both preterm and adult samples (Fig. 5J).

Further functional characterization of *S. epidermidis* ME 4 (consisting of 130 genes and 47 mapped proteins) using STRING analysis identified six protein clusters which were enriched in processes such as protoporphyrinogen IX biosynthesis and aromatic compound metabolism. One of these protein clusters in ME 4 consisted of three genes involved in heme biosynthesis (i.e., porphobilinogen deminase (*hemC*), uroporphyrinogen decarboxylase (*hemE*), and uroporphyrinogen-III synthase (*hemD/ABD30842.1*)) (Fig. 5I). *S. epidermidis* ME 3 (266 genes and 98 mapped proteins)  was enriched for molecular functions such as cation binding, metal ion binding, and catalytic activity, and showed elevated expression in term infant blood compared to preterm infant and adult blood samples (Fig. 5J).

The top 100 most highly expressed *S. epidermidis* and *S. aureus* genes across all host cohorts are shown in **Supplementary Tables 4 and 5**. In *S. epidermidis*, the most highly expressed genes included many hypothetical proteins and proteins involved in adhesion, fibrinogen-binding, and bacterial aggregation, whereas *S. aureus* gene expression was dominated by genes encoding ribosomal proteins, stress response factors, and iron uptake proteins, including components of the iron surface determinant (*Isd*) system.

Lastly, to further explore highly induced bacterial gene classes during blood challenge, the top 30 genes expressed by *S. epidermidis* and *S. aureus* in preterm and term infant blood samples were manually annotated into 12 functional categories based on literature and databases. Pie charts summarizing gene category distributions for each pathogen and host population are shown in Fig. 5E-H. This preliminary analysis showed a mostly comparable functional gene distribution in preterm and term infant blood by both bacterial species. However, more genes attributable to host interaction/virulence and iron uptake categories were observed in *S. epidermidis* in response to term infants, and a larger stress response was seen in both *S. aureus* and *S. epidermidis* in response to term infant blood.

## Discussion

CONS species, predominantly *S. epidermidis*, are the most frequently isolated LOS pathogens in very preterm infants^34,35^, yet the mechanisms driving their shift from commensal to pathogen are poorly understood. Moreover, the interplay between the preterm immune response and commensal virulence remains elusive, with current research often limited by the separation of host and pathogen responses. This study addresses these gaps by employing an ex vivo sepsis model and dual RNA-seq to simultaneously profile host leukocyte and staphylococcal transcriptional changes, providing new insights into pathogen-specific drivers of infection.

Our findings reveal distinct survival and proliferation patterns of *S. epidermidis* and *S. aureus* during a 90-minute blood challenge, highlighting divergent pathogenic strategies. Both our data, and previous research, suggest that while *S. aureus* employs active virulence and immune evasion strategies during infection, the low-virulence commensal *S. epidermidis* better fits the description of an “accidental” pathogen^30^, which focuses on stealth strategies instead of active toxicity and immune-countering responses upon displacement into host tissue. Notably, *S. aureus* colony counts significantly decreased only in term infant blood, suggesting a more effective blood-mediated bacterial killing capacity in this cohort compared to preterm infants. This underscores the capability of the term infant immune system to mount robust pro-inflammatory responses, despite being functionally distinct from the adult immune system, as it adapts to life outside the womb^36^. Importantly, all host cohorts significantly reduced *S. epidermidis* colony counts within the first 90 min of blood exposure. We hypothesize that preterm infants who develop late-onset sepsis (LOS) may possess distinct immune response deficiencies against *S. epidermidis*, setting them apart from the broader preterm population. This highlights the need for further investigation into immune mechanisms underlying the increased susceptibility to LOS in vulnerable neonates.

LOS triggers systemic immune-, metabolic-, and hematological responses in preterm infants^37,38^. Our data indicate that these changes occur rapidly upon bacterial stimulation, with preterm infant blood showing distinct activation of immune-metabolic pathways compared to term infants, particularly in response to *S. epidermidis*. Hypoxia- and coagulation-related processes were significantly upregulated in preterm infants exposed to *S. epidermidis*, suggesting pathogen-specific metabolic disruption. While the mechanisms of *S. epidermidis*-induced hypoxia remain unclear, prior studies link this pathogen to heightened susceptibility to perinatal hypoxic-ischemic brain injury in neonatal mice^39^. Consistent with this, a recent piglet model of preterm *S. epidermidis* infection (same strain 1457) revealed significant metabolic alterations in plasma and cerebrospinal fluid, including glycolysis, hypoxia, and disrupted cerebral energy metabolism^40^.

Both *S. epidermidis* and *S. aureus* stimulation elicited pro-inflammatory (e.g., IL-1) and anti-inflammatory (e.g., IL-4, IL-10) cytokine responses in preterm infant blood, mirroring profiles observed in clinical LOS cases^38^. Strikingly, preterm infants showed stronger cytokine responses to commensal *S. epidermidis* than to the more virulent *S. aureus*, possibly reflecting pathogen-induced metabolic stress. These early immune responses suggest that *S. epidermidis* infection may disrupt preterm metabolic homeostasis, exacerbating clinical outcomes.

Our data also indicate that *S. epidermidis* and *S. aureus* may adopt distinct iron acquisition strategies during blood challenge, reflecting their adaptation to iron-limited host environments^41^. In all cohorts, *S. aureus* highly expressed iron-scavenging *Isd*-family genes, indicating a preference for hemoglobin-bound heme uptake. Similarly, *S. epidermidis* upregulated siderophore genes, such as the ferrichrome ABC transporter, in term infant blood^42^. However, in preterm infant blood, *S. epidermidis* expressed multiple genes from the *hem* operon, suggesting an alternative strategy involving heme biosynthesis in nutrient-scarce conditions. This adaptation may exploit preterm infants’ reduced iron stores, lower hepcidin levels, and metabolic vulnerabilities, including reliance on exogenous glucose and impaired stress responses^43,44^. We hypothesize that *S. epidermidis* leverages host nutrient and iron deficiencies in preterm infants, inducing immune-metabolic dysregulation while upregulating essential metal biosynthesis pathways. These findings highlight the need for further investigation into iron metabolism and metabolic stress as potential factors in neonatal susceptibility to *S. epidermidis* infections.

## Limitations

This study utilised a blood challenge model with a defined challenge timepoint and bacterial inoculum. However, ex vivo sepsis models cannot fully recapitulate the complexity of clinical sepsis in vivo. The bacterial inoculum used in our model was higher than levels typically observed in clinical bacteraemia. This was necessary to elicit measurable host and bacterial transcriptional responses, and is consistent with prior ex vivo models^31,32,45^. We acknowledge that infection dynamics may vary between host cohorts, and longitudinal sampling will be essential to investigate temporal correlations between host and bacterial gene expression changes. As a hypothesis-generating pilot study, our sample size of eight was sufficient to identify cohort- and pathogen-specific transcriptional differences. However, future studies with larger sample sizes, multiple challenge timepoints, and greater cohort diversity will enhance our understanding of infection kinetics, account for potential confounding variables, and strengthen the generalizability of these findings. Future studies will ideally include samples from preterm infants < 28 weeks GA, which comprise the highest risk group for CONS sepsis. Finally, we recognise that transcriptional profiles do not always translate directly to protein-level changes and future work will benefit from integrating transcriptomics data with proteomic data and/or cytokine assays for a more comprehensive picture of host-pathogen interactions during sepsis.

## Conclusion

Our data suggest that the interplay between hosts and staphylococcal bacterial pathogens is highly complex and influenced not only by host factors, such as developmental age, but also by pathogen-specific virulence strategies employed during bacterial invasion of blood. Infection risk and poor sepsis outcomes in preterm infants may be driven by unique environmental and physiological risk factors specific to preterm infant population, as well as by an increased ability of LOS-causing *S. epidermidis* to exploit preterm infant nutritional deficiencies and immune-metabolic impairments. Simultaneous pro-inflammatory and immune-inhibitory signalling during infection may exacerbate immune dysregulation in the preterm population, particularly in response to commensal *S. epidermidis*. Future studies will benefit from larger sample sizes and longitudinal sampling in sepsis patients to further elucidate correlations in host-pathogen gene expression changes occurring during sepsis and identifying potential therapeutic targets and interventional pathways.

## Methods

### Ethics approvals and statement

The collection of blood samples from healthy adults at Murdoch University was approved by the Murdoch University Human Research Ethics Committee (2016/031). The Human Research Ethics Committee at King Edward Memorial Hospital for Women (KEMH), Perth, Western Australia, approved the blood sample collection from very preterm and term infants (RGS4774). Written informed consent was obtained either from study participants or parents/guardians prior to study participation. All research was performed in accordance with the Declaration of Helsinki, the Australian Code for the Responsible Conduct of Research 2018, and the National Statement on Ethical Conduct in Human Research.

### Study participants

8 adults (18–25 years), 8 very preterm infants (30–32 weeks GA) and 8 term infants (> 37 weeks GA) were recruited for this study over a period of 9 months (May 2022 to January 2023). Blood samples from all participants were processed within 120 min of blood collection and underwent bacterial challenge. Inclusion criteria for participating infants was informed consent by a parent/guardian and absence of concurrent infection and/or administration of systemic antibiotics in the previous 48 h. Relevant demographic data was collected from patient records. Inclusion criteria for adult study participants included informed consent, non-smokers, no history of dizziness, fainting or low blood pressure, no known bleeding disorder and/or taking blood-thinning medications, no medical conditions requiring recent medication, and not having received blood product or immunoglobulin in the past 3 months.

### Blood sample collection

Neonatal blood samples (1 ml) were collected by venepuncture into two Li-Heparin tubes on postnatal days 2–3 for term and days 7–11 for preterm infants. Healthy adult peripheral blood samples were collected by venepuncture into Li-Heparin tubes at Murdoch University.

### Blood challenge model

Mid-log bacterial cultures (~ 10^7^ CFU/ml) of *S. epidermidis* (WT 1457^46^) and *S. aureus* (ATCC 29523), were recovered from frozen (−80 °C) bead stocks onto horse blood agar (HBA) plates as needed, and incubated overnight at 37 °C. Single colonies were incubated in 10 ml sterile Trypticase Soy Broth (TSB) statically overnight at 37 °C to form a primary liquid culture. The following day, 20 ml of TSB was inoculated with primary cultures to reach an OD of ~ 0.06, and bacterial cultures were grown at 37 °C and 160 rpm until mid-logarithmic (mid-log) growth phase was reached. Bacterial stocks were transferred into sterile, nuclease-free 2 ml microcentrifuge tubes and washed twice with sterile phosphate-buffered saline (PBS) by centrifugation at 10,000 *x g* for 7 min to remove growth medium. Cell pellets were resuspended in 50 µl nuclease-free water (NFW) before 0.45 ml heparinised blood was added. Samples were carefully mixed and then incubated at 37 °C and 5% CO_2_ at 200 rpm for 90 min. The bacterial challenge dose and incubation period was selected based on prior optimisation experiments for ex vivo blood challenge modelling to analyse gene expression in host leukocytes and staphylococcal cells. The colony count (CFU/ml) of bacterial stocks and blood samples at timepoint 0 and post challenge was determined by spot plating on HBA plates. The remaining aliquot of blood (0.1 ml) from each donor was included as unstimulated control.

From each donor, 1 ml of blood was collected and divided into two 0.45 ml aliquots for bacterial challenge and a smaller 0.1 ml aliquot used as the unstimulated control. The reduced control volume was necessitated by ethical and practical constraints when sampling from very preterm infants (< 32 weeks GA), where maximum allowable blood draw volumes are strictly limited. While the lower volume yields fewer total cells, leukocyte distributions are preserved, ensuring that the control remains biologically valid for comparative analyses. This approach allowed us to maximise the information obtained from each participant while adhering to safe sampling limits in this vulnerable population.

After bacterial challenge, blood samples were stabilised with 2.76x PAXgene™ reagent (PreAnalytix; Qiagen/Becton Dickson) as previously described^38^ and frozen at −80 °C until further processing.

### RNA purification, RNA yield and integrity

A modified TRI reagent™ (Sigma) protocol was used to extract total RNA from PAXgene™-stabilised blood samples. Briefly, samples were spun down (3,200 *x g*, 10 min) and the supernatant was carefully removed. Cells were washed once with 1 ml NFW before pellets were dissolved in 100 µl NFW. 1 ml TRI reagent™ was added and samples were transferred into 2 ml bead-beating tubes containing 0.1 mm zirconia/silica beads (PureLink™ Invitrogen™). Cells were lysed by 3 × 1-min cycles of bead-beating at 3,000 rpm on a BioSpec Mini-Beadbeater-24 with 1 min breaks on ice. Samples were incubated at RT for 5 min before phase separation was performed by addition of 200 µl chloroform (MP Biomedical), followed by a 2 min incubation at RT and centrifugation at 12,000 *x g* for 15 min. RNA-containing aqueous phase was purified using the RNA Clean-and-Concentrator-25 kit (Zymo Research) according to the manufacturer’s instructions with a final elution volume of 30 µl. 40 U of RNaseOUT™ Recombinant Ribonuclease Inhibitor (40 U/µl, Invitrogen™) were added to eluted RNA samples before any remaining DNA traces were removed using the Ambion^®^ DNA-free™ DNase Treatment and Removal Reagents (Life Technologies) as per the manufacturer’s protocol.

### rRNA depletion, cDNA library preparation, RNA-sequencing

Dual species ribosomal RNA (rRNA) transcripts were depleted using the Illumina Ribo-Zero Plus RNA Depletion kit (Illumina) following the manufacturer’s guidelines. For cDNA conversion, an in-house adapted SmartSeq assay protocol^47^ using oligodT and random hexamer primers was employed. Full-length cDNAs were amplified, purified, and quantified using the Promega Quantus™ Fluorometer before the resulting double-stranded cDNA was prepared for sequencing using the NEBNext^®^ Ultra™ II FS DNA Library Prep Kit for Illumina (New England Biolabs). Deep sequencing of 72 samples was performed on a NovaSeq 6000 platform using 150 bp paired-end chemistry (Illumina).

### Bioinformatic analyses

#### Read processing and mapping

The raw sequencing reads were quality checked by FastQC v0.12.1^48^ and MultiQC v1.18^49^ following several criteria, including the sequencing quality score, GC content distribution, and base call quality. Potential contamination and sequencing adapter content were screened by evaluating over-presented sequences and using an in-house complete Illumina sequencing adaptor database. The QC analysis revealed that reads have been quality filtered by Illumina commercial software and no further filtering was performed, as further processing by Trimmomatic v0.39^50^ could not improve results. Bioinformatic rRNA filtering was performed using RiboDetector v0.2.8^51^, a Bi-directional Long Short-Term Memory (BiLSTM) neural network-based method, which was run on CPU mode with ensure parameter, which makes sure the classification has high confidence for paired-end reads. Host and bacterial read alignment was performed using bacterial whole genome references and annotation files for *S. epidermidis* 1457 (CP020463, chromosome; CP020462, plasmid^52^, *S. aureus* ATCC 29523 (CP009361, chromosome; CP009362, plasmid^53^ and human genome reference Hg38. Read mapping was performed using RNA-STAR v2.7.1a^54^ utilising a two-step approach. Briefly, in the first alignment, total filtered reads were mapped against the pathogen genomic references only. Reads that aligned in this first step were used for subsequent bacterial read analysis. In the second alignment, total reads were mapped to a combined references genome consisting of both host and bacterial genomic references. Reads that mapped to the human reference in this step were used for human read analysis. Gene counting/feature mapping was achieved by using RNA-STAR’s gene counting function and subread v2.0.6^55^ in pair-ended mode. Host and bacterial read count matrices were analysed separately in R (v4.3.1)^56^.

### RNA-seq data filtering

Globin RNA reads were removed bioinformatically as previously described^57^. Potentially influential outlier samples were identified using an RNA-seq data filtering approach recently described^58^, including analysis to quantify dissimilarity between samples using L1 distance and Tukey’s method, utilising Kolmogorov-Smirnov (K-S) test, and Hoeffding D statistic. Principal Component Analysis (PCA) was furthermore used to visually identify outlier samples. Samples were classified as outliers if flagged by more than one statistical method, consistent with recommended practice to distinguish technical artifacts (e.g., RNA degradation, library preparation issues) from true biological variation, which typically affects specific subsets of genes. Six host samples (five unstimulated adult samples and one unstimulated term infant sample) met these criteria and were removed prior to downstream analysis. PCA plots before and after outlier removal are provided in Supplementary Fig. 6, illustrating the impact of filtering on overall sample clustering. A Multiset Jaccard Index-based approach was used to filter low-count host genes. Before and after data filtering, gene-level quality control metrics were calculated and visualised.

### RNA-seq data normalisation

Filtered gene expression data was normalised and analysed using the *DESeq2* R package (v1.40.2). DESeq2 was selected because it is widely used and validated for RNA-seq count data, with robust performance in small cohorts and effective control of false discovery rates across a range of sample sizes and proportions of differentially expressed genes^59–61^. The *DESeq2* median-of-ratios method was used to normalise data prior to differential gene expression analysis, and variance-stabilised transformation was used to normalise data for PCA and Weighted Gene Co-Expression Network Analysis.

### Differential gene expression analysis

Preterm infant, term infant, and adult read count matrices were subset to perform differential gene expression analysis between unstimulated and pathogen-stimulated samples. Host genes with Benjamini-Hochberg (BH)-adjusted p-values < 0.05 and log2 fold changes (FC) of > 2, were identified as significantly differentially expressed genes (DEGs). Pathogen-induced significant DEGs in each cohort were then used to analyse differential gene expression patterns between the host cohorts using the same significance cut-offs. Bacterial differential gene expression analysis identified *S. epidermidis* and *S. aureus* DEGs in response to preterm infant, term infant, and adult whole blood based on BH-adjusted p-values < 0.05 and log2 FC of > 2.

### STRING protein association network analysis

Search Tool for the Retrieval of Interacting Genes (STRING) v12.0 (http://string-db.org/) was used to identify functional protein association networks for gene lists of interest. Markov Cluster Algorithm (MCL) was used with an inflation parameter of 3.0 and networks with connected nodes were based on high confidence scores (> 0.7). STRING database was used to perform pathway and gene set enrichment analysis on gene lists, including Gene Ontology (GO) term enrichment analysis for biological processes and molecular function, and Reactome pathway enrichment analysis.

### Gene set overrepresentation analysis

Overrepresentation analysis (ORA) was performed on host DEG lists using GO terms for biological processes. The *enrichGO* function from the *clusterProfiler* R package (v.4.8.3) was used to perform GO term enrichment analysis with a significance cut-off of p-value < 0.05 (Benjamini-Hochberg-corrected) and a q-value of < 0.10. UpSet plots were generated to visualise the overlap of GO terms across datasets using the *UpSetR* package (v1.40.2) in R.

### Weighted gene co-expression network analysis (WGCNA)

WGCNA was performed to identify co-expression modules in each cohort and sample stimulation condition using the *WGCNA* (v1.72-1) and *impute* (v1.74-1) R packages. For each analysis set, raw counts were rounded to integers and genes with total counts below 50 were removed. Expression values were then variance-stabilized with *DESeq2* and treated as expression input (dataIsExpr = TRUE). Soft-thresholding powers were selected separately for each dataset using the pickSoftThreshold function with Pearson correlation and a signed network, targeting a scale-free topology fit (R² ≥ 0.80). Networks were constructed with the blockwiseModules function using a signed topological overlap matrix, maximum block size of 5000, numeric labels, and a fixed random seed (1234). All other arguments were set to WGCNA defaults, including a minimum module size of 20 genes, deep split value of 2, dendrogram-respecting PAM stage, and merge cut height of 0.25. Module Eigengenes were calculated, and linear modelling was performed using the *lmFit* function from the *limma* (v3.56.2) R package with empirical Bayes smoothing. Multiple testing correction was applied, and statistics for module associations were obtained. Modules were ranked according to their differential expression across experimental variables of interest. A key was created to map genes to their respective modules and STRING protein network analysis was performed as described previously to identify associated proteins and functional enrichments in modules of interest.

### Data visualisation

Data were visualised using GraphPad Prism (v10.0.0, GraphPad Software) or R Studio (2023.06.1 + 524). Bar charts, PCA and multidimensional scaling (MDS) plots, and pie charts were created using the *ggplot2* (v3.4.4) and *stats* (v4.3.1) packages. The *vegan* (v2.6.4) package was used to create MDS plot ellipses. Heatmaps were created using either the *pheatmap* (v1.0.12) or ComplexHeatmap (v2.16.0) packages. Venn diagrams were prepared with the *VennDiagram* (v1.7.3) package. Gene set enrichment and gene set overrepresentation results are shown as dot and bar plots, which were created using the *enrichplot* (v1.20.3) package. Other R packages used for analysis include: *tidyverse* (v2.0.0), *reshape2* (v1.4.4), *dplyr* (v1.1.4), *gridExtra* (v2.3), and *org.Hs.eg.db* (v3.17.0) for human genome annotation.

### Statistical analysis

Shapiro-Wilk test was used to determine the normality of residuals (a = 0.05). Statistically significant difference between primary inoculum and post-challenge bacterial concentrations were determined by ratio paired t-test (preterm and term infant datasets) and non-parametric Wilcoxon signed rank test (adult dataset). Ordinary one-way ANOVA with Holm-Šidák multiple comparison’s test was used to compare normally distributed data, and Kruskal-Wallis test with Dunn’s multiple comparison test was used to compare non-parametric data (Fig. 1G-I). P-values: <0.05 (*), 0.01 (**), < 0.001 (***), < 0.0001 (****).

## Supplementary Information

Below is the link to the electronic supplementary material.

Supplementary Material 1

## Data Availability

Sequence data generated by this study are available on public data repositories of the European Molecular Biology Laboratory European Bioinformatics Institute (EMBL-EBI, ID: E-MTAB-14798) and can be accessed at [https://www.ebi.ac.uk/biostudies/arrayexpress/studies/E-MTAB-14798]. Processed count matrices, metadata, and all R Markdown scripts required to reproduce the analyses are available in the companion GitHub repository: https://github.com/iajoub/DUOS_RNAseq_Analysis.
