# Supplementary material for: Characterising commensal and pathogenic staphylococcal interactions with neonatal and adult blood
Source: Sci Rep. 2025 Dec 9;16:777. doi: 10.1038/s41598-025-30393-8 (PMC12779975; doi:10.1038/s41598-025-30393-8)
Supplement: Supplementary file 1 — Supplementary Material 1 [file 41598_2025_30393_MOESM1_ESM.docx]

## **Supplementary Data**

Table of contents:

*Supplementary Figures:*

- **Figure1:** Sample RNA yield (ng)
- **Figure 2:** Host Gene Set Overrepresentation Analysis.
- **Figure 3:** Heatmaps showing all Module Eigenenes (MEs) identified by Weighted Gene Co-expression Network Analysis (WGCNA) in blood in response to bacterial challenge (cohort-specific responses).
- **Figure 4:** Venn diagrams showing genes differentially expressed in the host cohorts in responses to *S. epidermidis* and *S. aureus.*
- **Figure 5:** Heatmaps showing all Module Eigenenes (MEs) identified by Weighted Gene Co-expression Network Analysis (WGCNA) in blood in response to bacterial challenge (pathogen-specific responses).

*Supplementary Tables:*

- **Table 1:** Total number and percentage of uniquely aligned host and bacterial reads in each RNA-seq sample.
- **Table 2:** Gene Ontology (GO) term enrichment for biological processes unique to preterm infants in response to *S. epidermidis*.
- **Table 3:** Gene Ontology (GO) term enrichment for biological processes unique to preterm infants in response to *S. aureus*.

**Supplementary Figure 1:** Total sample RNA yield from preterm infants (P), term infants (T), and adults (A), quantified from **A.** unstimulated, **B.** S. epidermidis-stimulated, and **C.** S. aureus-stimulated blood samples, respectively. Data shown are median + 95% CI, comparing total RNA yields obtained from preterm infant, term infant, and adult samples using Holm-Šídák’s multiple comparisons test with a single pooled variance (preterm and term infant data) and Dunn’s multiple comparison test (adult data)


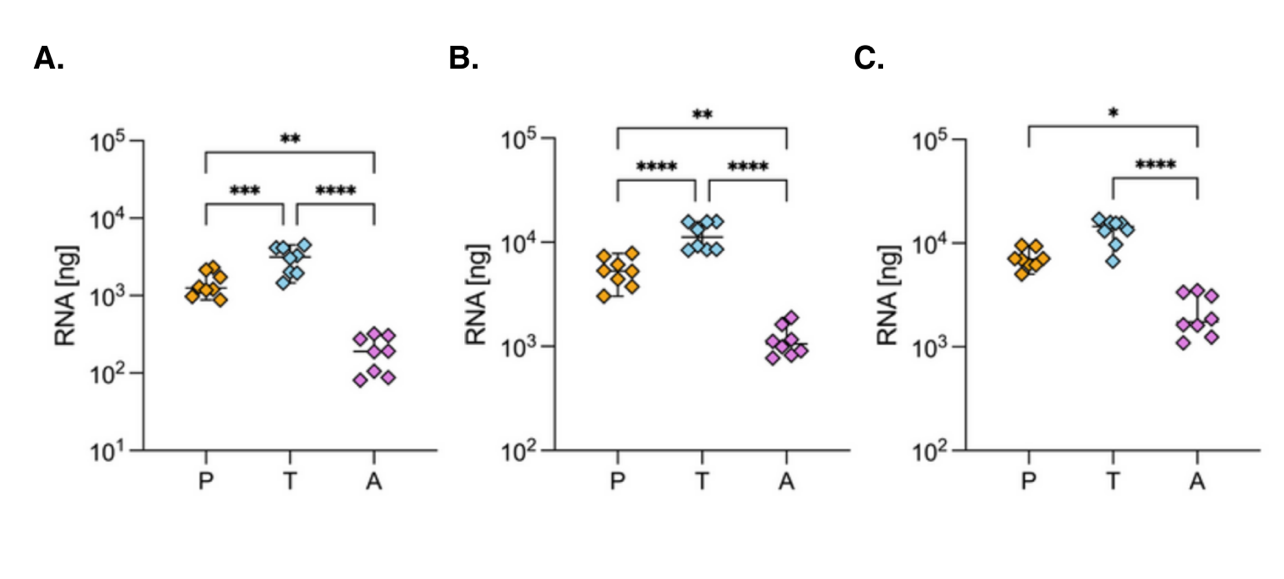


**Supplementary Figure 2:** Gene Set Overrepresentation Analysis was performed to identify enriched Gene Ontology (GO) terms of biological processes using the DEG list of pathogen-induced transcription changes across the 3 cohorts, sorted by gene count and coloured according to Benjamini-Hochberg-adjusted p-value (p.adjust). **A**. *S. epidermidis*-challenged preterm infant samples, **B.** *S. aureus*-challenged preterm infant samples, **C.** *S. epidermidis*-challenged term infant samples, **D.** *S. aureus*-challenged term infant samples, **E.** *S. epidermidis*-challenged adult samples, **F.** *S. aureus*-challenged adult samples.


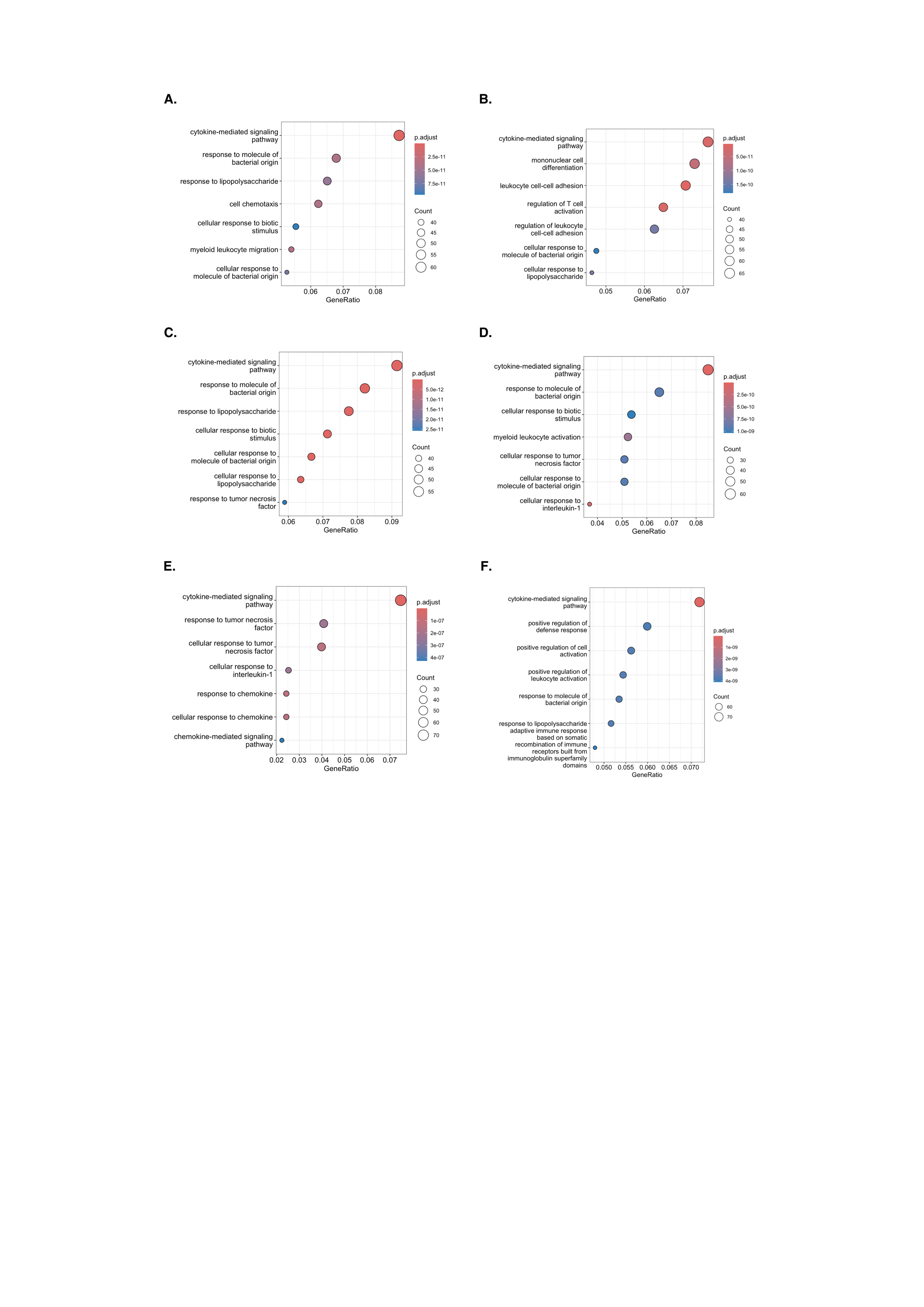


**Supplementary Figure 3:** Heatmaps showing all Module Eigenenes (MEs) identified by Weighted Gene Co-expression Network Analysis (WGCNA) in response to **A.** *S. epidermidis* and **B**. *S. aureus*.


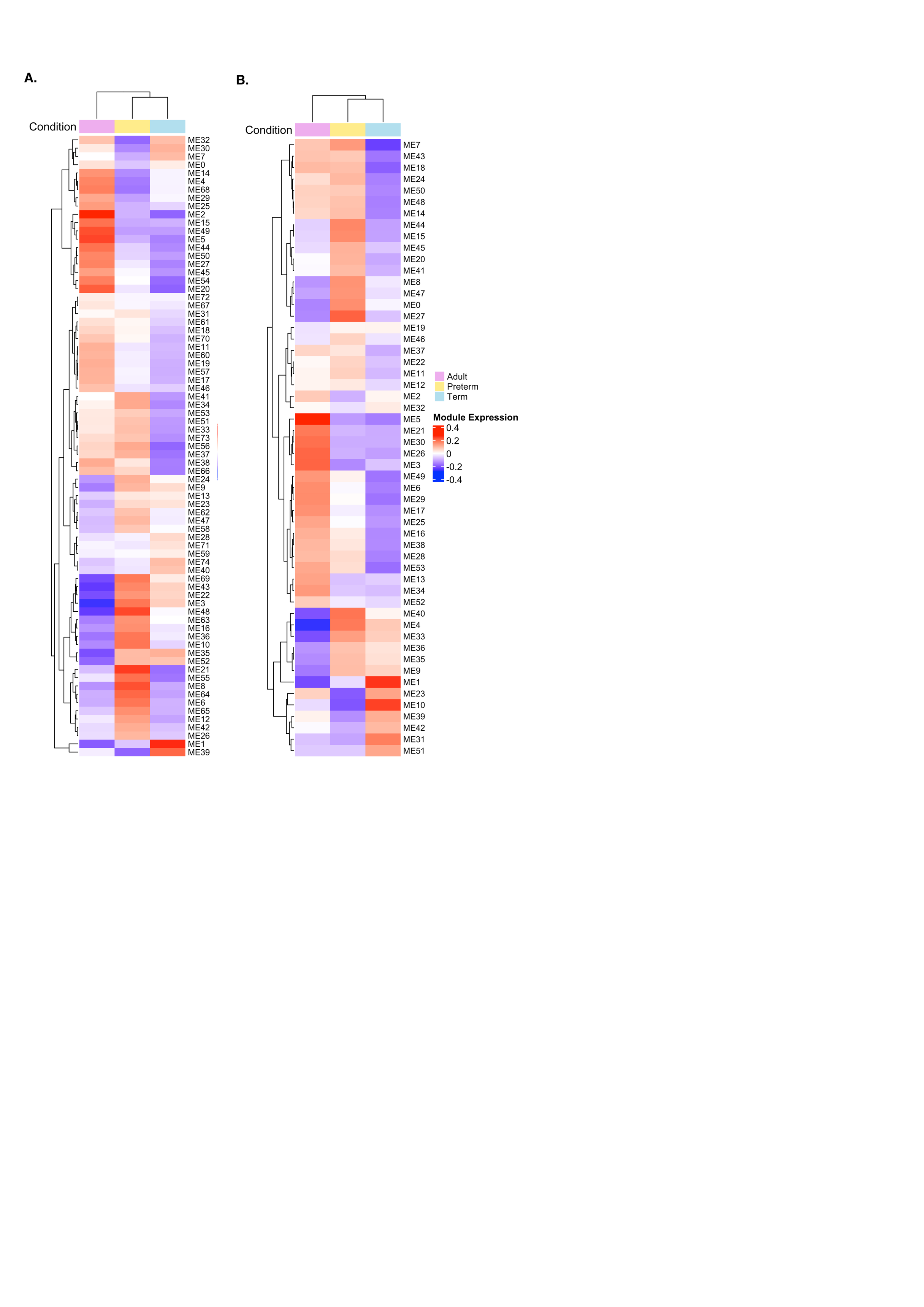


**Supplementary Figure 4:** Venn diagrams showing genes differentially expressed in **A.** preterm infants, **B**. term infant, and **C.** adults in responses to *S. epidermidis* and *S. aureus.*


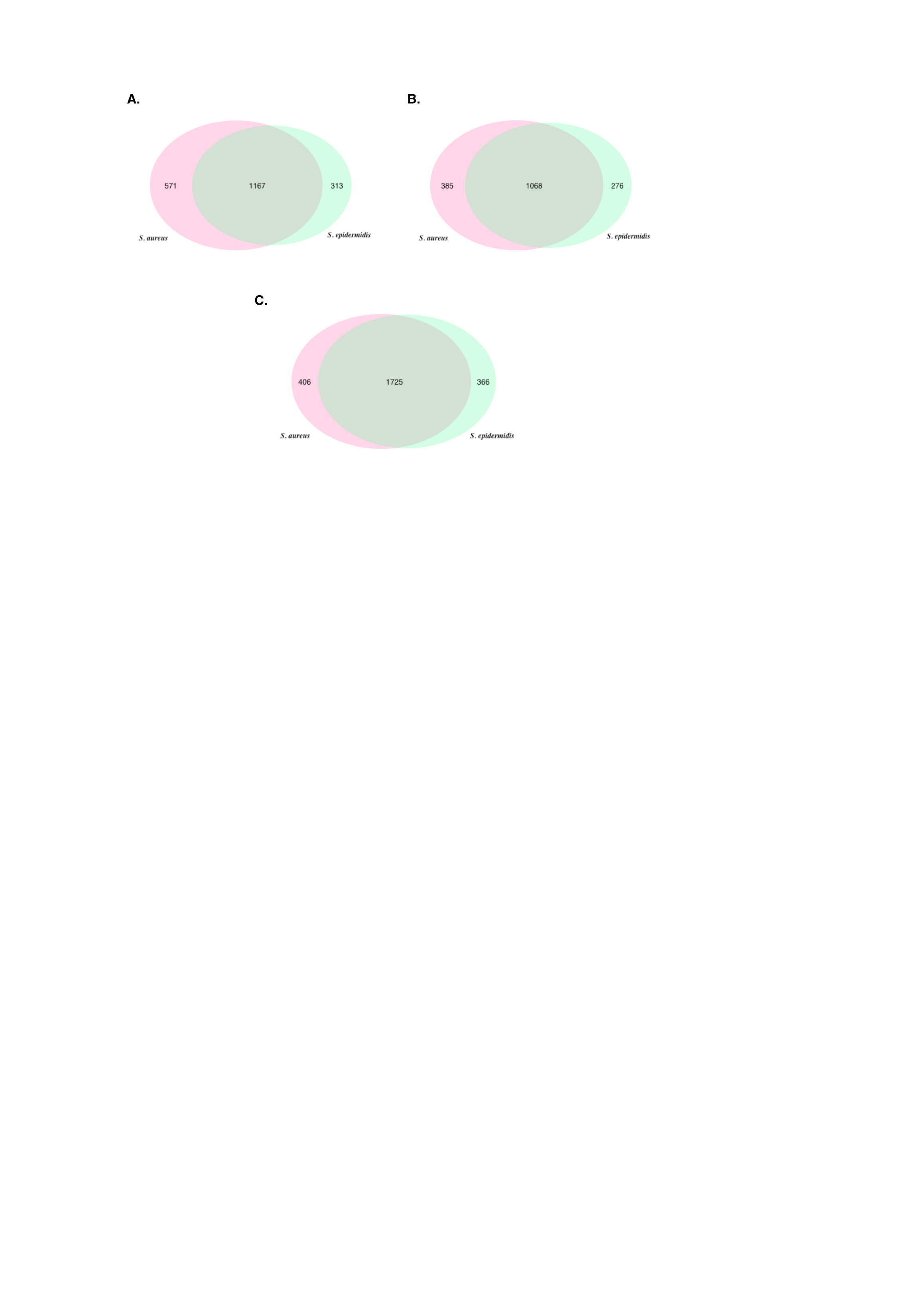


**Supplementary Figure 5:** Heatmaps showing all Module Eigenenes (MEs) identified by Weighted Gene Co-expression Network Analysis (WGCNA) in **A.** preterm infants and **B.** term infants and their expression across conditions (unstimulated, *S. epidermidis*- and *S. aureus*-stimulated samples).

**
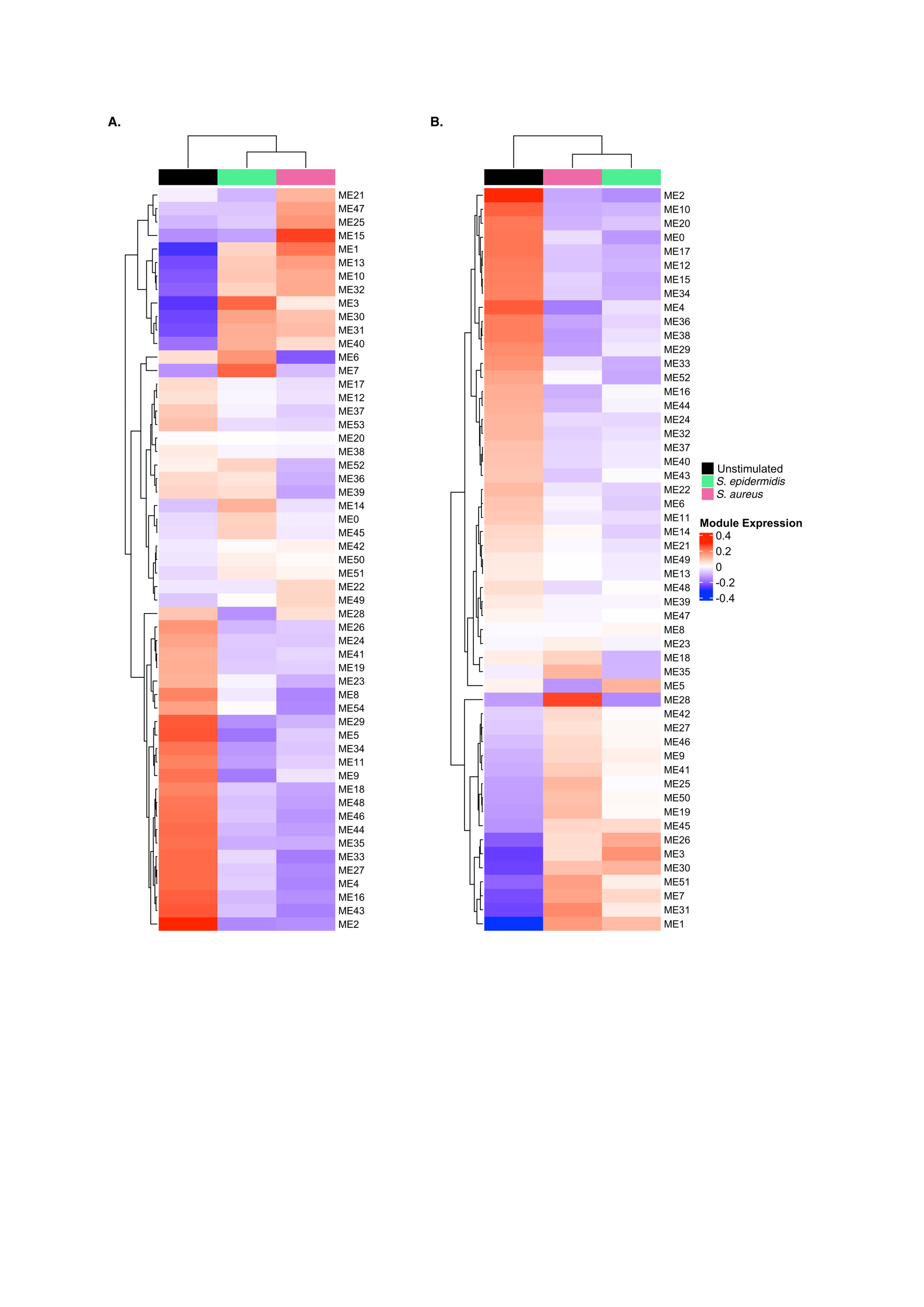
**

**Supplementary Figure 6: Principal component analysis (PCA) plots before and after sample outlier removal.** PCA plots of host RNA-seq data before (top row) and after (bottom row) sample filtering. Each point represents an individual sample, coloured and shaped by cohort and stimulation condition (A = adult, T = term infant, P = preterm infant; US = unstimulated, SE = S. epidermidis-stimulated, SA = S. aureus-stimulated). Ellipses denote 95% confidence intervals for each group. Outlier detection was performed using a multi-step filtering pipeline (L1 distance, Tukey’s method, Kolmogorov–Smirnov test, Hoeffding’s D statistic), with samples classified as outliers if flagged by more than one method. Five unstimulated adult samples and one unstimulated term infant sample were removed prior to downstream analyses. Post-filtering PCA demonstrates improved within-group clustering and clearer separation of cohorts and stimulation conditions.

**
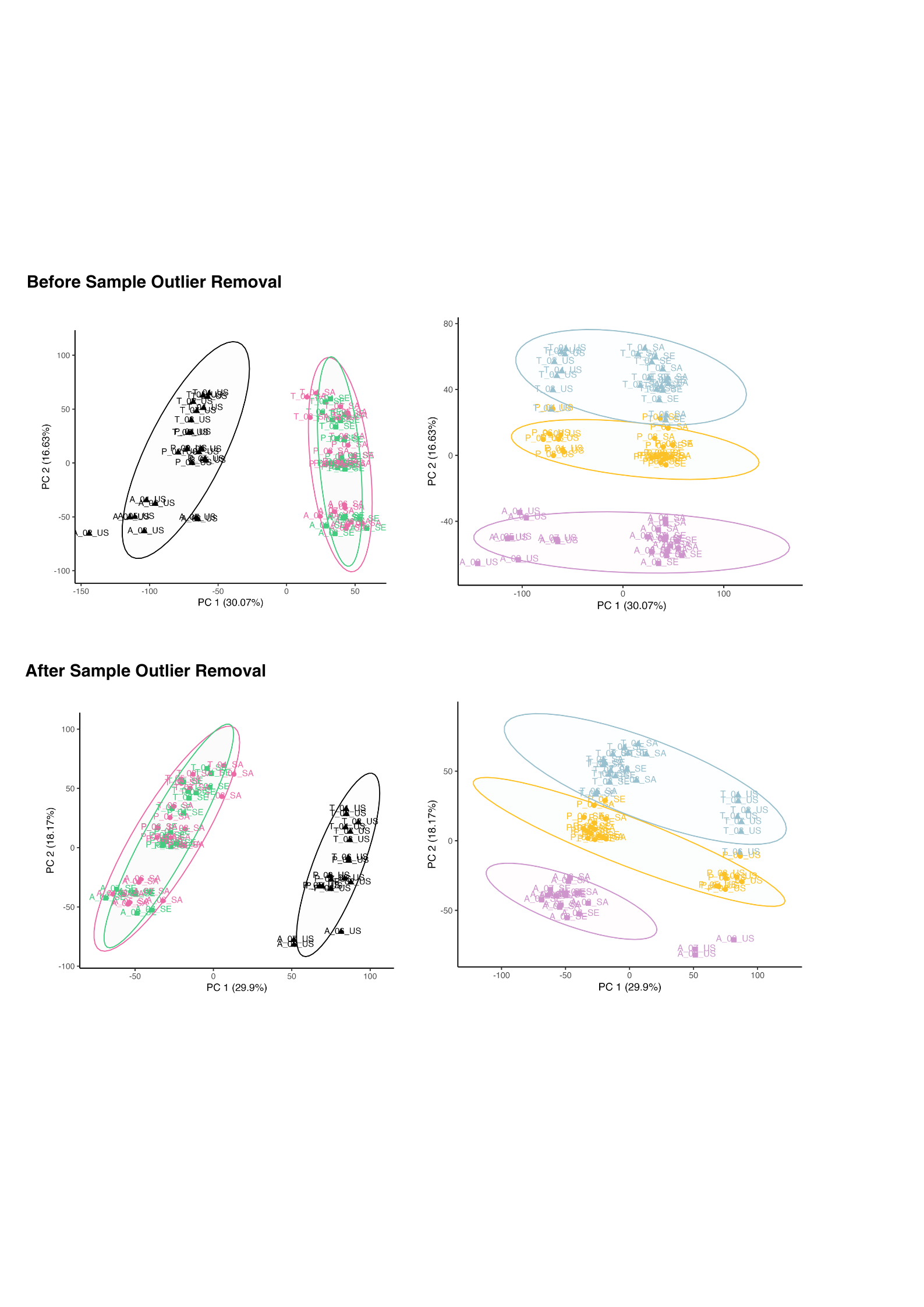
**

**Supplementary Table 1:** Total number and percentage of uniquely aligned host and bacterial reads in each RNA-seq sample.

| **Sample** | **TotalFiltered** | **UniqueAlign _Host** | **UniqueAlign _Pathogen** | **MultipleAlign** | **Unmapped** | **Total _UniqueAlign** | **Percentagen _UniqueAlign** | **Precentage _MultiMapped** |
| --- | --- | --- | --- | --- | --- | --- | --- | --- |
| **P01_US** | 22,274,485 | 15,345,920 | NA | 2,984,122 | 2,984,122 | 15,345,920 | 68.9% | 13.4% |
| **P02_US** | 21,333,327 | 14,770,552 | NA | 2,803,995 | 2,803,995 | 14,770,552 | 69.2% | 13.1% |
| **P03_US** | 22,835,868 | 15,265,771 | NA | 3,208,813 | 3,208,813 | 15,265,771 | 66.8% | 14.1% |
| **P04_US** | 21,545,178 | 14,243,155 | NA | 4,003,372 | 4,003,372 | 14,243,155 | 66.1% | 18.6% |
| **P05_US** | 25,837,536 | 17,195,191 | NA | 3,250,592 | 3,250,592 | 17,195,191 | 66.6% | 12.6% |
| **P06_US** | 19,498,729 | 13,381,702 | NA | 2,683,016 | 2,683,016 | 13,381,702 | 68.6% | 13.8% |
| **P07_US** | 25,857,265 | 16,536,483 | NA | 3,923,254 | 3,923,254 | 16,536,483 | 64.0% | 15.2% |
| **P08_US** | 15,675,984 | 9,554,623 | NA | 2,339,024 | 2,339,024 | 9,554,623 | 61.0% | 14.9% |
| **T01_US** | 22,809,872 | 16,388,898 | NA | 2,292,850 | 2,292,850 | 16,388,898 | 71.9% | 10.1% |
| **T02_US** | 18,917,903 | 12,626,681 | NA | 2,943,461 | 2,943,461 | 12,626,681 | 66.7% | 15.6% |
| **T03_US** | 19,506,351 | 13,802,665 | NA | 2,238,242 | 2,238,242 | 13,802,665 | 70.8% | 11.5% |
| **T04_US** | 17,863,315 | 12,434,787 | NA | 2,273,998 | 2,273,998 | 12,434,787 | 69.6% | 12.7% |
| **T05_US** | 64,563,358 | 50,619,550 | NA | 7,341,243 | 7,341,243 | 50,619,550 | 78.4% | 11.4% |
| **T06_US** | 20,297,604 | 14,816,943 | NA | 2,314,375 | 2,314,375 | 14,816,943 | 73.0% | 11.4% |
| **T07_US** | 21,558,728 | 15,403,503 | NA | 2,467,253 | 2,467,253 | 15,403,503 | 71.4% | 11.4% |
| **T08_US** | 22,345,849 | 16,030,940 | NA | 2,516,539 | 2,516,539 | 16,030,940 | 71.7% | 11.3% |
| **A01_US** | 53,285,885 | 37,335,212 | NA | 7,989,298 | 7,989,298 | 37,335,212 | 70.1% | 15.0% |
| **A02_US** | 48,213,635 | 26,258,017 | NA | 6,917,184 | 6,917,184 | 26,258,017 | 54.5% | 14.3% |
| **A03_US** | 56,800,280 | 40,484,713 | NA | 9,984,442 | 9,984,442 | 40,484,713 | 71.3% | 17.6% |
| **A04_US** | 57,279,866 | 40,235,956 | NA | 8,365,806 | 8,365,806 | 40,235,956 | 70.2% | 14.6% |
| **A05_US** | 54,552,096 | 36,009,305 | NA | 7,356,104 | 7,356,104 | 36,009,305 | 66.0% | 13.5% |
| **A06_US** | 24,623,769 | 17,228,618 | NA | 3,761,587 | 3,761,587 | 17,228,618 | 70.0% | 15.3% |
| **A07_US** | 18,840,311 | 12,538,705 | NA | 2,817,201 | 2,817,201 | 12,538,705 | 66.6% | 15.0% |
| **A08_US** | 21,092,880 | 14,695,668 | NA | 2,794,705 | 2,794,705 | 14,695,668 | 69.7% | 13.2% |
| **P01_SE** | 26,458,393 | 18,653,630 | 345,848 | 3,910,445 | 3,548,470 | 18,999,478 | 71.8% | 14.8% |
| **P02_SE** | 15,671,734 | 10,710,296 | 342,552 | 2,330,414 | 2,288,472 | 11,052,848 | 70.5% | 14.9% |
| **P03_SE** | 18,301,498 | 12,325,244 | 359,930 | 3,008,342 | 2,607,982 | 12,685,174 | 69.3% | 16.4% |
| **P04_SE** | 22,616,017 | 14,512,847 | 348,613 | 4,142,161 | 3,612,396 | 14,861,460 | 65.7% | 18.3% |
| **P05_SE** | 20,318,738 | 13,733,410 | 354,239 | 3,064,790 | 3,166,299 | 14,087,649 | 69.3% | 15.1% |
| **P06_SE** | 16,413,053 | 10,168,336 | 405,187 | 2,623,241 | 3,216,289 | 10,573,523 | 64.4% | 16.0% |
| **P07_SE** | 20,593,532 | 12,998,604 | 462,930 | 2,955,867 | 4,176,131 | 13,461,534 | 65.4% | 14.4% |
| **P08_SE** | 19,200,299 | 13,083,909 | 372,000 | 3,051,454 | 2,692,936 | 13,455,909 | 70.1% | 15.9% |
| **T01_SE** | 18,979,978 | 12,995,281 | 340,788 | 2,268,653 | 3,375,256 | 13,336,069 | 70.3% | 12.0% |
| **T02_SE** | 18,710,046 | 11,927,593 | 318,041 | 3,155,919 | 3,308,493 | 12,245,634 | 65.4% | 16.9% |
| **T03_SE** | 18,698,743 | 12,748,751 | 333,213 | 2,652,303 | 2,964,476 | 13,081,964 | 70.0% | 14.2% |
| **T04_SE** | 16,522,729 | 11,467,891 | 229,836 | 2,126,730 | 2,698,272 | 11,697,727 | 70.8% | 12.9% |
| **T05_SE** | 21,854,728 | 15,613,344 | 379,600 | 2,854,999 | 3,006,785 | 15,992,944 | 73.2% | 13.1% |
| **T06_SE** | 22,885,906 | 15,239,225 | 533,874 | 3,160,953 | 3,951,854 | 15,773,099 | 68.9% | 13.8% |
| **T07_SE** | 19,228,564 | 12,768,286 | 362,048 | 2,669,998 | 3,428,232 | 13,130,334 | 68.3% | 13.9% |
| **T08_SE** | 21,621,622 | 14,929,782 | 528,347 | 2,710,858 | 3,452,635 | 15,458,129 | 71.5% | 12.5% |
| **A01_SE** | 21,066,162 | 14,862,247 | 318,013 | 2,898,021 | 2,987,881 | 15,180,260 | 72.1% | 13.8% |
| **A02_SE** | 42,132,324 | 29,305,539 | 463,174 | 6,890,343 | 5,473,268 | 29,768,713 | 70.7% | 16.4% |
| **A03_SE** | 21,833,341 | 14,945,951 | 411,507 | 2,809,510 | 3,666,373 | 15,357,458 | 70.3% | 12.9% |
| **A04_SE** | 26,967,498 | 18,464,332 | 559,610 | 3,651,224 | 4,292,332 | 19,023,942 | 70.5% | 13.5% |
| **A05_SE** | 22,417,452 | 15,700,501 | 380,590 | 2,929,215 | 3,407,146 | 16,081,091 | 71.7% | 13.1% |
| **A06_SE** | 22,417,452 | 15,700,501 | 366,731 | 2,949,009 | 3,401,211 | 16,067,232 | 71.7% | 13.2% |
| **A07_SE** | 17,897,082 | 11,769,379 | 637,511 | 2,494,036 | 2,996,156 | 12,406,890 | 69.3% | 13.9% |
| **A08_SE** | 22,419,832 | 15,461,754 | 645,946 | 3,094,097 | 3,218,035 | 16,107,700 | 71.8% | 13.8% |
| **P01_SA** | 20,843,850 | 11,497,961 | 2,891,250 | 2,370,028 | 4,084,611 | 14,389,211 | 69.0% | 11.4% |
| **P02_SA** | 17,274,736 | 8,277,413 | 3,600,242 | 2,236,753 | 3,160,328 | 11,877,655 | 68.8% | 12.9% |
| **P03_SA** | 17,005,363 | 9,420,256 | 1,531,606 | 2,418,410 | 3,635,091 | 10,951,862 | 64.4% | 14.2% |
| **P04_SA** | 19,663,603 | 10,552,295 | 2,335,036 | 2,885,832 | 3,890,440 | 12,887,331 | 65.5% | 14.7% |
| **P05_SA** | 18,498,184 | 11,436,040 | 1,251,330 | 2,587,105 | 3,223,709 | 12,687,370 | 68.6% | 14.0% |
| **P06_SA** | 17,382,215 | 10,467,762 | 1,150,186 | 2,594,249 | 3,170,018 | 11,617,948 | 66.8% | 14.9% |
| **P07_SA** | 18,870,311 | 9,545,300 | 1,364,403 | 2,633,305 | 5,327,303 | 10,909,703 | 57.8% | 14.0% |
| **P08_SA** | 13,001,994 | 3,882,858 | 3,801,701 | 1,432,011 | 3,885,424 | 7,684,559 | 59.1% | 11.0% |
| **T01_SA** | 23,498,469 | 14,120,399 | 2,616,676 | 2,318,106 | 4,443,288 | 16,737,075 | 71.2% | 9.9% |
| **T02_SA** | 19,804,204 | 12,476,043 | 1,216,172 | 3,042,770 | 3,069,219 | 13,692,215 | 69.1% | 15.4% |
| **T03_SA** | 18,636,129 | 11,596,699 | 971,098 | 2,601,704 | 3,466,628 | 12,567,797 | 67.4% | 14.0% |
| **T04_SA** | 31,858,972 | 18,608,815 | 1,552,410 | 5,857,719 | 5,840,028 | 20,161,225 | 63.3% | 18.4% |
| **T05_SA** | 19,168,681 | 12,670,656 | 1,140,296 | 2,380,538 | 2,977,191 | 13,810,952 | 72.0% | 12.4% |
| **T06_SA** | 19,733,421 | 12,238,267 | 1,569,126 | 2,541,846 | 3,384,182 | 13,807,393 | 70.0% | 12.9% |
| **T07_SA** | 17,132,040 | 9,723,374 | 2,152,639 | 2,011,347 | 3,244,680 | 11,876,013 | 69.3% | 11.7% |
| **T08_SA** | 19,929,774 | 11,987,066 | 2,270,958 | 2,245,022 | 3,426,728 | 14,258,024 | 71.5% | 11.3% |
| **A01_SA** | 23,518,601 | 15,641,489 | 881,871 | 3,141,732 | 3,853,509 | 16,523,360 | 70.3% | 13.4% |
| **A02_SA** | 22,236,525 | 15,170,487 | 921,352 | 3,104,042 | 3,040,644 | 16,091,839 | 72.4% | 14.0% |
| **A03_SA** | 21,304,408 | 13,400,374 | 1,292,959 | 2,978,182 | 3,632,893 | 14,693,333 | 69.0% | 14.0% |
| **A04_SA** | 18,008,100 | 11,408,322 | 1,095,041 | 2,437,184 | 3,067,553 | 12,503,363 | 69.4% | 13.5% |
| **A05_SA** | 22,322,368 | 13,319,548 | 2,508,245 | 2,804,858 | 3,689,717 | 15,827,793 | 70.9% | 12.6% |
| **A06_SA** | 19,215,431 | 12,683,327 | 509,084 | 2,853,559 | 3,169,461 | 13,192,411 | 68.7% | 14.9% |
| **A07_SA** | 27,117,720 | 7,791,101 | 9,666,103 | 2,816,535 | 6,843,981 | 17,457,204 | 64.4% | 10.4% |
| **A08_SA** | 22,561,967 | 7,296,467 | 8,311,649 | 2,279,808 | 4,674,043 | 15,608,116 | 69.2% | 10.1% |

**Supplementary Table 2:** Gene Ontology (GO) term enrichment for biological processes unique to preterm infants in response to *S. epidermidis*. Count: Number of genes identified as part of a GO term in gene list; p.adjust: Benjamini-Hochberg-corrected p-value; q-value: p-value adjusted for the false discovery rate (FDR).

| **Go term ID** | **Description** | **Count** | **p.adjust** | **qvalue** |
| --- | --- | --- | --- | --- |
| GO:0001667 | ameboidal-type cell migration | 33 | 0.021 | 0.016 |
| GO:0198738 | cell-cell signaling by wnt | 32 | 0.015 | 0.012 |
| GO:0016055 | Wnt signaling pathway | 31 | 0.023 | 0.018 |
| GO:0051960 | regulation of nervous system development | 31 | 0.023 | 0.018 |
| GO:0070482 | response to oxygen levels | 23 | 0.044 | 0.035 |
| GO:0048608 | reproductive structure development | 22 | 0.032 | 0.025 |
| GO:0061458 | reproductive system development | 22 | 0.035 | 0.027 |
| GO:0001666 | response to hypoxia | 21 | 0.043 | 0.033 |
| GO:0051146 | striated muscle cell differentiation | 21 | 0.047 | 0.037 |
| GO:0032868 | response to insulin | 20 | 0.040 | 0.031 |
| GO:0051048 | negative regulation of secretion | 15 | 0.037 | 0.029 |
| GO:0009266 | response to temperature stimulus | 15 | 0.042 | 0.033 |
| GO:0030324 | lung development | 15 | 0.046 | 0.036 |
| GO:0046683 | response to organophosphorus | 14 | 0.008 | 0.006 |
| GO:0014074 | response to purine-containing compound | 14 | 0.016 | 0.013 |
| GO:0060041 | retina development in camera-type eye | 14 | 0.028 | 0.022 |
| GO:1903531 | negative regulation of secretion by cell | 14 | 0.029 | 0.023 |
| GO:0010976 | positive regulation of neuron projection development | 14 | 0.030 | 0.023 |
| GO:0014902 | myotube differentiation | 13 | 0.016 | 0.012 |
| GO:0006986 | response to unfolded protein | 13 | 0.032 | 0.025 |
| GO:0030183 | B cell differentiation | 13 | 0.049 | 0.039 |
| GO:0046425 | regulation of receptor signaling pathway via JAK-STAT | 12 | 0.011 | 0.008 |
| GO:0035914 | skeletal muscle cell differentiation | 11 | 0.002 | 0.001 |
| GO:0051591 | response to cAMP | 11 | 0.009 | 0.007 |
| GO:0043406 | positive regulation of MAP kinase activity | 11 | 0.045 | 0.035 |
| GO:0071887 | leukocyte apoptotic process | 11 | 0.045 | 0.035 |
| GO:0051851 | modulation by host of symbiont process | 10 | 0.016 | 0.013 |
| GO:0090502 | RNA phosphodiester bond hydrolysis, endonucleolytic | 10 | 0.021 | 0.017 |
| GO:0045453 | bone resorption | 9 | 0.011 | 0.008 |
| GO:0007422 | peripheral nervous system development | 9 | 0.026 | 0.021 |
| GO:0061097 | regulation of protein tyrosine kinase activity | 9 | 0.037 | 0.029 |
| GO:0098586 | cellular response to virus | 9 | 0.044 | 0.035 |
| GO:0002088 | lens development in camera-type eye | 9 | 0.049 | 0.038 |
| GO:0060324 | face development | 8 | 0.011 | 0.009 |
| GO:0099024 | plasma membrane invagination | 8 | 0.019 | 0.015 |
| GO:0010324 | membrane invagination | 8 | 0.032 | 0.025 |
| GO:0038034 | signal transduction in absence of ligand | 8 | 0.036 | 0.028 |
| GO:0042490 | mechanoreceptor differentiation | 8 | 0.036 | 0.028 |
| GO:0097192 | extrinsic apoptotic signaling pathway in absence of ligand | 8 | 0.036 | 0.028 |
| GO:0014015 | positive regulation of gliogenesis | 8 | 0.042 | 0.033 |
| GO:0050922 | negative regulation of chemotaxis | 8 | 0.044 | 0.034 |
| GO:2000379 | positive regulation of reactive oxygen species metabolic process | 8 | 0.044 | 0.034 |
| GO:0072678 | T cell migration | 8 | 0.047 | 0.036 |
| GO:0060325 | face morphogenesis | 7 | 0.003 | 0.002 |
| GO:0060323 | head morphogenesis | 7 | 0.008 | 0.006 |
| GO:0048246 | macrophage chemotaxis | 7 | 0.012 | 0.009 |
| GO:1905521 | regulation of macrophage migration | 7 | 0.017 | 0.013 |
| GO:0061098 | positive regulation of protein tyrosine kinase activity | 7 | 0.018 | 0.014 |
| GO:0006911 | phagocytosis, engulfment | 7 | 0.026 | 0.021 |
| GO:0045761 | regulation of adenylate cyclase activity | 7 | 0.031 | 0.024 |
| GO:0101023 | vascular endothelial cell proliferation | 7 | 0.033 | 0.026 |
| GO:1905562 | regulation of vascular endothelial cell proliferation | 7 | 0.033 | 0.026 |
| GO:1902930 | regulation of alcohol biosynthetic process | 7 | 0.036 | 0.028 |
| GO:1905523 | positive regulation of macrophage migration | 6 | 0.007 | 0.005 |
| GO:1900101 | regulation of endoplasmic reticulum unfolded protein response | 6 | 0.012 | 0.009 |
| GO:0032350 | regulation of hormone metabolic process | 6 | 0.032 | 0.025 |
| GO:0045840 | positive regulation of mitotic nuclear division | 6 | 0.046 | 0.036 |
| GO:2000249 | regulation of actin cytoskeleton reorganization | 6 | 0.049 | 0.039 |
| GO:0060008 | Sertoli cell differentiation | 5 | 0.013 | 0.010 |
| GO:0045932 | negative regulation of muscle contraction | 5 | 0.022 | 0.017 |
| GO:0043153 | entrainment of circadian clock by photoperiod | 5 | 0.025 | 0.019 |
| GO:0046885 | regulation of hormone biosynthetic process | 5 | 0.025 | 0.019 |
| GO:0090023 | positive regulation of neutrophil chemotaxis | 5 | 0.025 | 0.019 |
| GO:0045672 | positive regulation of osteoclast differentiation | 5 | 0.028 | 0.022 |
| GO:0010758 | regulation of macrophage chemotaxis | 5 | 0.035 | 0.027 |
| GO:0030194 | positive regulation of blood coagulation | 5 | 0.035 | 0.027 |
| GO:0071624 | positive regulation of granulocyte chemotaxis | 5 | 0.035 | 0.027 |
| GO:1900048 | positive regulation of hemostasis | 5 | 0.035 | 0.027 |
| GO:0009648 | photoperiodism | 5 | 0.039 | 0.031 |
| GO:0050820 | positive regulation of coagulation | 5 | 0.044 | 0.034 |
| GO:0045762 | positive regulation of adenylate cyclase activity | 5 | 0.048 | 0.037 |
| GO:0060674 | placenta blood vessel development | 5 | 0.048 | 0.037 |
| GO:0060009 | Sertoli cell development | 4 | 0.019 | 0.014 |
| GO:0045986 | negative regulation of smooth muscle contraction | 4 | 0.023 | 0.018 |
| GO:1900102 | negative regulation of endoplasmic reticulum unfolded protein response | 4 | 0.023 | 0.018 |
| GO:0036005 | response to macrophage colony-stimulating factor | 4 | 0.032 | 0.025 |
| GO:0097396 | response to interleukin-17 | 4 | 0.032 | 0.025 |
| GO:0097398 | cellular response to interleukin-17 | 4 | 0.032 | 0.025 |
| GO:1901163 | regulation of trophoblast cell migration | 4 | 0.032 | 0.025 |
| GO:0010885 | regulation of cholesterol storage | 4 | 0.037 | 0.029 |
| GO:0061450 | trophoblast cell migration | 4 | 0.037 | 0.029 |
| GO:0010759 | positive regulation of macrophage chemotaxis | 4 | 0.044 | 0.034 |
| GO:0010801 | negative regulation of peptidyl-threonine phosphorylation | 4 | 0.044 | 0.034 |
| GO:0010878 | cholesterol storage | 4 | 0.049 | 0.039 |
| GO:0030223 | neutrophil differentiation | 3 | 0.044 | 0.035 |
| GO:0032353 | negative regulation of hormone biosynthetic process | 3 | 0.044 | 0.035 |

**Supplementary Table 3:** Gene Ontology (GO) term enrichment for biological processes unique to preterm infants in response to *S. aureus*. Count: Number of genes identified as part of a GO term in gene list; p.adjust: Benjamini-Hochberg-corrected p-value; q-value: p-value adjusted for the false discovery rate (FDR).

| **Go term ID** | **Description** | **Count** | **p.adjust** | **qvalue** |
| --- | --- | --- | --- | --- |
| GO:0010975 | regulation of neuron projection development | 41 | 0.001 | 0.001 |
| GO:0051960 | regulation of nervous system development | 36 | 0.021 | 0.016 |
| GO:0051962 | positive regulation of nervous system development | 26 | 0.010 | 0.008 |
| GO:0001763 | morphogenesis of a branching structure | 22 | 0.004 | 0.003 |
| GO:0050769 | positive regulation of neurogenesis | 21 | 0.029 | 0.023 |
| GO:0042445 | hormone metabolic process | 21 | 0.041 | 0.033 |
| GO:0022604 | regulation of cell morphogenesis | 21 | 0.046 | 0.037 |
| GO:0061138 | morphogenesis of a branching epithelium | 20 | 0.008 | 0.006 |
| GO:0046777 | protein autophosphorylation | 20 | 0.043 | 0.034 |
| GO:0010976 | positive regulation of neuron projection development | 17 | 0.014 | 0.011 |
| GO:0010952 | positive regulation of peptidase activity | 17 | 0.050 | 0.040 |
| GO:0032963 | collagen metabolic process | 16 | 0.001 | 0.001 |
| GO:0035051 | cardiocyte differentiation | 16 | 0.034 | 0.027 |
| GO:0030177 | positive regulation of Wnt signaling pathway | 15 | 0.033 | 0.026 |
| GO:0055007 | cardiac muscle cell differentiation | 14 | 0.026 | 0.021 |
| GO:1901222 | regulation of NIK/NF-kappaB signaling | 12 | 0.046 | 0.037 |
| GO:0055013 | cardiac muscle cell development | 11 | 0.022 | 0.018 |
| GO:0060021 | roof of mouth development | 11 | 0.033 | 0.026 |
| GO:0070664 | negative regulation of leukocyte proliferation | 11 | 0.045 | 0.036 |
| GO:0010586 | miRNA metabolic process | 11 | 0.047 | 0.038 |
| GO:0002275 | myeloid cell activation involved in immune response | 11 | 0.050 | 0.040 |
| GO:0042446 | hormone biosynthetic process | 10 | 0.014 | 0.011 |
| GO:0070373 | negative regulation of ERK1 and ERK2 cascade | 10 | 0.032 | 0.026 |
| GO:2000628 | regulation of miRNA metabolic process | 10 | 0.040 | 0.032 |
| GO:0030574 | collagen catabolic process | 9 | 0.004 | 0.003 |
| GO:0046636 | negative regulation of alpha-beta T cell activation | 9 | 0.004 | 0.003 |
| GO:0045058 | T cell selection | 9 | 0.008 | 0.007 |
| GO:0002820 | negative regulation of adaptive immune response | 9 | 0.022 | 0.018 |
| GO:0000768 | syncytium formation by plasma membrane fusion | 9 | 0.025 | 0.020 |
| GO:0140253 | cell-cell fusion | 9 | 0.025 | 0.020 |
| GO:1901224 | positive regulation of NIK/NF-kappaB signaling | 9 | 0.036 | 0.029 |
| GO:0035914 | skeletal muscle cell differentiation | 9 | 0.039 | 0.031 |
| GO:0014015 | positive regulation of gliogenesis | 9 | 0.042 | 0.033 |
| GO:0045600 | positive regulation of fat cell differentiation | 9 | 0.048 | 0.038 |
| GO:0032350 | regulation of hormone metabolic process | 8 | 0.006 | 0.005 |
| GO:0006953 | acute-phase response | 8 | 0.019 | 0.015 |
| GO:0036230 | granulocyte activation | 8 | 0.019 | 0.015 |
| GO:0010761 | fibroblast migration | 8 | 0.036 | 0.029 |
| GO:0061756 | leukocyte adhesion to vascular endothelial cell | 8 | 0.043 | 0.034 |
| GO:0043616 | keratinocyte proliferation | 8 | 0.046 | 0.037 |
| GO:0046885 | regulation of hormone biosynthetic process | 7 | 0.002 | 0.002 |
| GO:0010743 | regulation of macrophage derived foam cell differentiation | 7 | 0.009 | 0.007 |
| GO:0010742 | macrophage derived foam cell differentiation | 7 | 0.020 | 0.016 |
| GO:0043368 | positive T cell selection | 7 | 0.020 | 0.016 |
| GO:0090077 | foam cell differentiation | 7 | 0.022 | 0.018 |
| GO:0010762 | regulation of fibroblast migration | 7 | 0.025 | 0.020 |
| GO:0046633 | alpha-beta T cell proliferation | 7 | 0.049 | 0.039 |
| GO:0042832 | defense response to protozoan | 6 | 0.017 | 0.013 |
| GO:0001562 | response to protozoan | 6 | 0.022 | 0.018 |
| GO:0002360 | T cell lineage commitment | 6 | 0.025 | 0.020 |
| GO:0097066 | response to thyroid hormone | 6 | 0.025 | 0.020 |
| GO:0009072 | aromatic amino acid metabolic process | 6 | 0.032 | 0.026 |
| GO:0002828 | regulation of type 2 immune response | 6 | 0.037 | 0.029 |
| GO:0000289 | nuclear-transcribed mRNA poly(A) tail shortening | 6 | 0.041 | 0.033 |
| GO:0050869 | negative regulation of B cell activation | 6 | 0.041 | 0.033 |
| GO:2000515 | negative regulation of CD4-positive, alpha-beta T cell activation | 6 | 0.041 | 0.033 |
| GO:0001893 | maternal placenta development | 6 | 0.046 | 0.037 |
| GO:0051968 | positive regulation of synaptic transmission, glutamatergic | 6 | 0.046 | 0.037 |
| GO:0060999 | positive regulation of dendritic spine development | 6 | 0.046 | 0.037 |
| GO:0070528 | protein kinase C signaling | 6 | 0.046 | 0.037 |
| GO:0001660 | fever generation | 5 | 0.002 | 0.002 |
| GO:0032306 | regulation of prostaglandin secretion | 5 | 0.005 | 0.004 |
| GO:0032308 | positive regulation of prostaglandin secretion | 5 | 0.005 | 0.004 |
| GO:0009074 | aromatic amino acid family catabolic process | 5 | 0.009 | 0.007 |
| GO:0031649 | heat generation | 5 | 0.012 | 0.010 |
| GO:0002827 | positive regulation of T-helper 1 type immune response | 5 | 0.015 | 0.012 |
| GO:0032305 | positive regulation of icosanoid secretion | 5 | 0.026 | 0.021 |
| GO:0032303 | regulation of icosanoid secretion | 5 | 0.030 | 0.024 |
| GO:0034104 | negative regulation of tissue remodeling | 5 | 0.030 | 0.024 |
| GO:0043371 | negative regulation of CD4-positive, alpha-beta T cell differentiation | 5 | 0.030 | 0.024 |
| GO:0072170 | metanephric tubule development | 5 | 0.036 | 0.029 |
| GO:0060143 | positive regulation of syncytium formation by plasma membrane fusion | 5 | 0.041 | 0.033 |
| GO:0001779 | natural killer cell differentiation | 5 | 0.047 | 0.037 |
| GO:2000193 | positive regulation of fatty acid transport | 5 | 0.047 | 0.037 |
| GO:0006568 | tryptophan metabolic process | 4 | 0.016 | 0.013 |
| GO:0031650 | regulation of heat generation | 4 | 0.021 | 0.017 |
| GO:0032351 | negative regulation of hormone metabolic process | 4 | 0.021 | 0.017 |
| GO:0033083 | regulation of immature T cell proliferation | 4 | 0.021 | 0.017 |
| GO:0033084 | regulation of immature T cell proliferation in thymus | 4 | 0.021 | 0.017 |
| GO:0006586 | indolalkylamine metabolic process | 4 | 0.026 | 0.021 |
| GO:0051132 | NK T cell activation | 4 | 0.026 | 0.021 |
| GO:0060213 | positive regulation of nuclear-transcribed mRNA poly(A) tail shortening | 4 | 0.026 | 0.021 |
| GO:0010713 | negative regulation of collagen metabolic process | 4 | 0.032 | 0.026 |
| GO:0010421 | hydrogen peroxide-mediated programmed cell death | 4 | 0.040 | 0.032 |
| GO:0032725 | positive regulation of granulocyte macrophage colony-stimulating factor production | 4 | 0.040 | 0.032 |
| GO:0060211 | regulation of nuclear-transcribed mRNA poly(A) tail shortening | 4 | 0.040 | 0.032 |

**Supplementary Table 4**: Top 100 expressed genes by *S. epidermidis* in response to blood challenge across all host cohorts. Locus tags are specific to *S. epidermidis* strain 1457. Protein product and RefSeq reference numbers are based on sequence homology with S*. epidermidis* strain ATCC 12228.

| **Locus_tag** | **Product** | **RefSeq homology** |
| --- | --- | --- |
| B4U56_00055 | hypothetical protein | WP_002470106.1 |
| B4U56_00115 | DNA gyrase subunit A | NP_763560.1 |
| B4U56_00200 | hypothetical protein | NP_763575.1 |
| B4U56_00225 | YSIRK signal domain/LPXTG anchor domain surface protein | WP_016898462.1 |
| B4U56_00415 | arginine-ornithine antiporter | NP_763660.1 |
| B4U56_00650 | Accumulation-associated protein | NP_763730.1 |
| B4U56_00665 | adenosylmethionine--8-amino-7-oxononanoate transaminase | NP_763735.1 |
| B4U56_00665 | NA | NA |
| B4U56_00725 | flavocytochrome c | NP_763750.1 |
| B4U56_00770 | nickel ABC transporter, nickel/metallophore periplasmic binding protein | NP_763760.1 |
| B4U56_00955 | non-ribosomal peptide synthetase | NP_763798.1 |
| B4U56_00965 | triacylglycerol lipase | NP_763800.1 |
| B4U56_01005 | dihydrolipoyl dehydrogenase | NP_763808.1 |
| B4U56_01010 | ABC transporter substrate-binding protein | NP_763809.1 |
| B4U56_01020 | branched-chain alpha-keto acid dehydrogenase subunit E2 | NP_763811.1 |
| B4U56_01140 | lipase | NP_763836.1 |
| B4U56_01220 | adhesin | WP_000044537.1 |
| B4U56_01320 | PTS mannose transporter subunit IIABC | NP_765783.1 |
| B4U56_01375 | ornithine carbamoyltransferase | NP_765771.1 |
| B4U56_01430 | NA | NA |
| B4U56_01430 | hypothetical protein | NP_765759.1 |
| B4U56_01485 | ABC transporter ATP-binding protein | NP_765748.1 |
| B4U56_01550 | sulfite reductase [NADPH] flavoprotein, alpha-component | NP_765735.1 |
| B4U56_01985 | gamma-glutamyltranspeptidase | NP_765644.1 |
| B4U56_02095 | hypothetical protein | NP_765621.1 |
| B4U56_02230 | NgoFVII family restriction endonuclease | WP_002470135.1 |
| B4U56_02375 | MFS transporter | NP_765565.1 |
| B4U56_02535 | nitrate reductase subunit alpha | NP_765530.1 |
| B4U56_02830 | hypothetical protein | NP_765470.1 |
| B4U56_03025 | CHAP domain-containing protein | NP_765431.1 |
| B4U56_03275 | DNA topoisomerase 3 | NP_765383.1 |
| B4U56_03540 | alkaline shock protein 23 | NP_765329.1 |
| B4U56_03565 | iron citrate ABC transporter substrate-binding protein | NP_765323.1 |
| B4U56_03680 | hypothetical protein | NP_765308.1 |
| B4U56_04330 | YSIRK signal domain/LPXTG anchor domain surface protein | WP_010959210.1 |
| B4U56_04445 | NAD(+) synthetase | NP_765151.1 |
| B4U56_04925 | DNA repair protein Rad50 | NP_765079.1 |
| B4U56_05235 | leucine--tRNA ligase | NP_764986.1 |
| B4U56_05245 | NA | NA |
| B4U56_05245 | YSIRK signal domain/LPXTG anchor domain surface protein | NP_764984.1 |
| B4U56_05315 | DNA translocase FtsK | NP_764969.1 |
| B4U56_05380 | D-3-phosphoglycerate dehydrogenase | NP_764956.1 |
| B4U56_05505 | acetyl-CoA carboxylase carboxyl transferase subunit alpha | NP_764930.1 |
| B4U56_05525 | citrate synthase | NP_764926.1 |
| B4U56_05535 | DNA-binding response regulator | NP_764924.1 |
| B4U56_05685 | valine--tRNA ligase | NP_764894.1 |
| B4U56_05880 | RecD/TraA family helicase | NP_764857.1 |
| B4U56_05885 | alanine--tRNA ligase | NP_764856.1 |
| B4U56_06055 | molecular chaperone DnaK | NP_764822.1 |
| B4U56_06290 | glycine dehydrogenase | NP_764775.1 |
| B4U56_06735 | NA | NA |
| B4U56_06735 | hyperosmolarity resistance protein Ebh | WP_002489985.1 |
| B4U56_06860 | 2-oxoglutarate dehydrogenase subunit E1 | NP_764652.1 |
| B4U56_07190 | aconitate hydratase | NP_764587.1 |
| B4U56_07205 | nuclease SbcCD subunit C | NP_764584.1 |
| B4U56_07270 | catalase | NP_764571.1 |
| B4U56_07375 | hypothetical protein | NP_764549.1 |
| B4U56_07450 | glycerol kinase | NP_764533.1 |
| B4U56_07570 | DNA translocase FtsK | NP_764508.1 |
| B4U56_07630 | DNA polymerase III subunit alpha | WP_001251777.1 |
| B4U56_07700 | DNA topoisomerase 1 | WP_002453083.1 |
| B4U56_07745 | hypothetical protein | NP_764472.1 |
| B4U56_07785 | chromosome segregation protein SMC | NP_764464.1 |
| B4U56_07890 | primosomal protein N' | NP_764443.1 |
| B4U56_07935 | carbamoyl-phosphate synthase large chain | WP_002442460.1 |
| B4U56_07990 | isoleucine--tRNA ligase | NP_764423.1 |
| B4U56_08175 | phenylalanine--tRNA ligase subunit beta | NP_764387.1 |
| B4U56_08185 | RNA methyltransferase | NP_764385.1 |
| B4U56_08195 | cell surface protein | WP_002475943.1 |
| B4U56_08215 | hypothetical protein | NP_764380.1 |
| B4U56_08275 | pyruvate carboxylase | NP_764368.1 |
| B4U56_08415 | cytochrome D ubiquinol oxidase subunit II | NP_764340.1 |
| B4U56_08550 | cytochrome ubiquinol oxidase subunit I | WP_001010769.1 |
| B4U56_08590 | bifunctional autolysin | NP_764305.1 |
| B4U56_08740 | peptidase M14 | WP_017464459.1 |
| B4U56_08755 | phage tail tape measure protein | WP_002500094.1 |
| B4U56_08755 | NA | NA |
| B4U56_09190 | peptide ABC transporter substrate-binding protein | NP_764239.1 |
| B4U56_09240 | chaperone protein ClpB | NP_764229.1 |
| B4U56_09285 | helicase-exonuclease AddAB subunit AddA | NP_764219.1 |
| B4U56_09630 | Organic hydroperoxide resistance protein-like 1 | NP_764146.1 |
| B4U56_09830 | DUF4887 domain-containing protein | NP_764109.1 |
| B4U56_09935 | preprotein translocase subunit SecA | NP_764090.1 |
| B4U56_10085 | bifunctional acetaldehyde-CoA/alcohol dehydrogenase | NP_764061.1 |
| B4U56_10115 | ABC transporter ATP-binding protein | NP_764055.1 |
| B4U56_10125 | lipoteichoic acid synthase | NP_764049.1 |
| B4U56_10305 | thiol reductant ABC exporter subunit CydC | NP_764011.1 |
| B4U56_10605 | cation:proton antiporter | NP_763952.1 |
| B4U56_10930 | MSCRAMM family adhesin SdrE | WP_002505000.1 |
| B4U56_10930 | NA | NA |
| B4U56_11020 | elongation factor Tu | NP_763867.1 |
| B4U56_11025 | elongation factor G | WP_017804675.1 |
| B4U56_11040 | ribosomal protein L7Ae | NP_763863.1 |
| B4U56_11045 | DNA-directed RNA polymerase subunit beta' | NP_763862.1 |
| B4U56_11050 | DNA-directed RNA polymerase subunit beta | NP_763861.1 |
| B4U56_11065 | 50S ribosomal protein L10 | WP_001837303.1 |
| B4U56_11140 | DNA repair protein RadA | NP_763843.1 |
| B4U56_11145 | ATP-dependent Clp protease ATP-binding subunit ClpC | NP_763842.1 |
| B4U56_11270 | lysine--tRNA ligase | WP_002436798.1 |
| B4U56_11340 | transcription-repair coupling factor | NP_765835.1 |
| B4U56_11520 | glutamate synthase subunit alpha | NP_765867.1 |
| B4U56_11625 | hypothetical protein | NP_765887.1 |
| B4U56_11715 | xanthine permease | WP_000793018.1 |
| B4U56_11990 | YSIRK signal domain/LPXTG anchor domain surface protein | NP_765950.1 |
| B4U56_12030 | lipase | NP_765958.1 |
| B4U56_12100 | tRNA uridine-5-carboxymethylaminomethyl(34) synthesis GTPase MnmE | NP_765972.1 |

**Supplementary Table 5**: Top 100 expressed genes by *S. aureus* in response to blood challenge across all host cohorts. Locus-tags are specific to *S. aureus* strain ATCC 29523.

| **Locus_tag** | **Product** | **RefSeq/SwissProt homology** |
| --- | --- | --- |
| KQ76_00295 | peptigoglycan-binding protein LysM | WP_000728711.1 |
| KQ76_00315 | iron ABC transporter substrate-binding protein | WP_001045125.1 |
| KQ76_01615 | single-stranded DNA-binding protein | WP_020808019.1 |
| KQ76_02380 | zinc metalloprotease | WP_001167893.1 |
| KQ76_02530 | CtsR family transcriptional regulator | Q2YSD9.1 |
| KQ76_02535 | excinuclease ABC subunit B | WP_000882074.1 |
| KQ76_02545 | Clp protease ClpX | WP_000897133.1 |
| KQ76_02610 | 50S ribosomal protein L11 | Q6GBV0.1 |
| KQ76_02620 | 50S ribosomal protein L10 | WP_001651981.1 |
| KQ76_02635 | DNA-directed RNA polymerase subunit beta | WP_000918669.1 |
| KQ76_02640 | DNA-directed RNA polymerase subunit beta' | WP_020808074.1 |
| KQ76_02645 | ribosomal protein L7Ae | WP_020444636.1 |
| KQ76_02650 | 30S ribosomal protein S12 | Q6GBU2.1 |
| KQ76_02655 | 30S ribosomal protein S7 | WP_013054923.1 |
| KQ76_02660 | elongation factor G | WP_017804675.1 |
| KQ76_02665 | elongation factor Tu | WP_001040566.1 |
| KQ76_02735 | hydrolase | WP_000934494.1 |
| KQ76_03005 | hypothetical protein | WP_001826078.1 |
| KQ76_03095 | manganese ABC transporter substrate-binding protein | WP_000737654.1 |
| KQ76_03100 | manganese ABC transporter permease | WP_001576039.1 |
| KQ76_03490 | histidine kinase | Q6GIT7.1 |
| KQ76_03495 | response regulator SaeR | Q5HHW4.1 |
| KQ76_03560 | glycerol phosphate lipoteichoic acid synthase | WP_001626656.1 |
| KQ76_03635 | ribonucleotide-diphosphate reductase subunit alpha | WP_000855513.1 |
| KQ76_03750 | preprotein translocase subunit SecA | WP_000506532.1 |
| KQ76_04220 | alanine-phosphoribitol ligase | WP_000129644.1 |
| KQ76_04440 | chaperone protein ClpB | WP_001619681.1 |
| KQ76_04495 | peptide ABC transporter permease | WP_002871020.1 |
| KQ76_04515 | peptide ABC transporter substrate-binding protein | WP_001584759.1 |
| KQ76_05010 | quinol oxidase subunit 2 | Q2YX14.1 |
| KQ76_05165 | ribonuclease J | Q2YX35.1 |
| KQ76_05190 | pyruvate dehydrogenase | WP_002467471.1 |
| KQ76_05195 | 2-oxoisovalerate dehydrogenase | WP_001819806.1 |
| KQ76_05200 | branched-chain alpha-keto acid dehydrogenase subunit E2 | WP_000863434.1 |
| KQ76_05240 | chitinase | WP_000145497.1 |
| KQ76_05270 | GTP-binding protein TypA | WP_000182652.1 |
| KQ76_05360 | DNA-binding protein | WP_000872155.1 |
| KQ76_05375 | heme transporter IsdB | WP_001041575.1 |
| KQ76_05380 | heme transporter IsdA | WP_000160847.1 |
| KQ76_05510 | fibrinogen-binding protein | WP_000739212.1 |
| KQ76_05680 | cell division protein FtsA | WP_000391037.1 |
| KQ76_05780 | orotate phosphoribosyltransferase | WP_001825439.1 |
| KQ76_05995 | succinyl-CoA synthetase subunit beta | WP_006190114.1 |
| KQ76_06020 | DNA topoisomerase I | Q5HGI2.1 |
| KQ76_06040 | ATP-dependent protease | WP_001788685.1 |
| KQ76_06055 | 30S ribosomal protein S2 | P66544.1 |
| KQ76_06160 | cell division protein FtsK | WP_000035750.1 |
| KQ76_06210 | ribonuclease | WP_000926578.1 |
| KQ76_06870 | ABC transporter ATP-binding protein | WP_001802897.1 |
| KQ76_06975 | dihydrolipoamide succinyltransferase | WP_001115449.1 |
| KQ76_07110 | matrix-binding protein | WP_001109387.1 |
| KQ76_07195 | Holliday junction resolvase | WP_001796863.1 |
| KQ76_07365 | peptidoglycan-binding protein LysM | WP_000069309.1 |
| KQ76_07890 | 6-phosphogluconate dehydrogenase | Q931R3.1 |
| KQ76_08210 | iron transporter | WP_006190421.1 |
| KQ76_08215 | hypothetical protein | Q7A5C5.1 |
| KQ76_08250 | molecular chaperone DnaK | WP_000034719.1 |
| KQ76_08255 | heat shock protein GrpE | Q5HFH9.1 |
| KQ76_08260 | HrcA family transcriptional regulator | P68792.1 |
| KQ76_08430 | Holliday junction resolvase | P67490.1 |
| KQ76_08680 | glutamyl-tRNA reductase | Q6GG33.1 |
| KQ76_08695 | trigger factor | WP_000127583.1 |
| KQ76_08720 | 50S ribosomal protein L20 | Q8CS77.1 |
| KQ76_08730 | translation initiation factor IF-3 | P65140.1 |
| KQ76_08805 | isocitrate dehydrogenase | P99167.1 |
| KQ76_08810 | citrate synthase | WP_001662816.1 |
| KQ76_08860 | universal stress protein UspA | WP_017638225.1 |
| KQ76_08885 | universal stress protein UspA | WP_000634176.1 |
| KQ76_09635 | DNA repair protein Rad50 | WP_001836286.1 |
| KQ76_10160 | adenylosuccinate lyase | WP_001607912.1 |
| KQ76_10330 | phosphodiesterase | WP_000283439.1 |
| KQ76_10520 | histidine kinase | Q6GF36.1 |
| KQ76_10800 | RNA helicase | WP_001178930.1 |
| KQ76_10855 | membrane protein | WP_015581891.1 |
| KQ76_10935 | ATP F0F1 synthase subunit alpha | WP_017636769.1 |
| KQ76_10945 | ATP F0F1 synthase subunit B | WP_000140676.1 |
| KQ76_11040 | fructose-bisphosphate aldolase | WP_001662541.1 |
| KQ76_11190 | glucosamine--fructose-6-phosphate aminotransferase | WP_000334461.1 |
| KQ76_11345 | iron citrate ABC transporter substrate-binding protein | WP_001214650.1 |
| KQ76_11375 | alkaline shock protein 23 | WP_002464378.1 |
| KQ76_11380 | membrane protein | WP_002464379.1 |
| KQ76_11385 | hypothetical protein | WP_000825821.1 |
| KQ76_11540 | 50S ribosomal protein L13 | WP_001819530.1 |
| KQ76_11570 | DNA-directed RNA polymerase subunit alpha | Q49ZE2.1 |
| KQ76_11580 | 30S ribosomal protein S13 | P66388.1 |
| KQ76_11600 | preprotein translocase subunit SecY | WP_020444777.1 |
| KQ76_11640 | 50S ribosomal protein L5 | Q4L8A2.1 |
| KQ76_11645 | 50S ribosomal protein L24 | WP_002461784.1 |
| KQ76_11685 | 50S ribosomal protein L2 | P60430.1 |
| KQ76_11695 | 50S ribosomal protein L4 | WP_002467756.1 |
| KQ76_11700 | 50S ribosomal protein L3 | A5IV34.1 |
| KQ76_11705 | 30S ribosomal protein S10 | Q931G5.1 |
| KQ76_11890 | ferrichrome ABC transporter substrate-binding protein | WP_000735038.1 |
| KQ76_12630 | immunoglobulin-binding protein sbi | WP_001827136.1 |
| KQ76_12640 | gamma-hemolysin subunit A | WP_000594524.1 |
| KQ76_13360 | 1-pyrroline-5-carboxylate dehydrogenase | WP_000259691.1 |
| KQ76_13620 | malate:quinone oxidoreductase | WP_001130049.1 |
| KQ76_13785 | clumping factor B | WP_000745926.1 |
| KQ76_14165 | hypothetical protein | WP_000790915.1 |
| KQ76_14265 | ArsR family transcriptional regulator | P30338.1 |
| KQ76_00295 | peptigoglycan-binding protein LysM | WP_000728711.1 |
| KQ76_00315 | iron ABC transporter substrate-binding protein | WP_001045125.1 |
| KQ76_01615 | single-stranded DNA-binding protein | WP_020808019.1 |
| KQ76_02380 | zinc metalloprotease | WP_001167893.1 |
| KQ76_02530 | CtsR family transcriptional regulator | Q2YSD9.1 |
| KQ76_02535 | excinuclease ABC subunit B | WP_000882074.1 |
